# Supramolecular Peptide Assemblies as Antimicrobial Scaffolds

**DOI:** 10.3390/molecules25122751

**Published:** 2020-06-14

**Authors:** Andrew W. Simonson, Matthew R. Aronson, Scott H. Medina

**Affiliations:** 1 Department of Biomedical Engineering, The Pennsylvania State University, Suite 122, CBE Building, University Park, PA 16802-4400, USA; aws5784@psu.edu (A.W.S.); mja5575@psu.edu (M.R.A.); 2 Huck Institutes of the Life Sciences, The Pennsylvania State University, University Park, PA 16802-4400, USA

**Keywords:** self-assembly, peptides, antimicrobial, multidrug resistance, drug delivery

## Abstract

Antimicrobial discovery in the age of antibiotic resistance has demanded the prioritization of non-conventional therapies that act on new targets or employ novel mechanisms. Among these, supramolecular antimicrobial peptide assemblies have emerged as attractive therapeutic platforms, operating as both the bactericidal agent and delivery vector for combinatorial antibiotics. Leveraging their programmable inter- and intra-molecular interactions, peptides can be engineered to form higher ordered monolithic or co-assembled structures, including nano-fibers, -nets, and -tubes, where their unique bifunctionalities often emerge from the supramolecular state. Further advancements have included the formation of macroscopic hydrogels that act as bioresponsive, bactericidal materials. This systematic review covers recent advances in the development of supramolecular antimicrobial peptide technologies and discusses their potential impact on future drug discovery efforts.

## 1. Introduction

Antibiotics have been a pillar of medicine since ancient times, and an essential tool in modern healthcare since the discovery of penicillin in 1928 [1,2]. With their widespread use, antibiotics have been largely credited with the rapid elongation of life expectancy in the years following. However, the ease of access and pervasive use of these ‘miracle’ compounds has exposed several clinical hurdles, with no obvious solutions. The most well-established, of course, is the emergence of multidrug-resistance (MDR) through the avoidance of antibiotic action via microbial dormancy and altered metabolism [3,4], mutational alteration of the drug target [5,6], and over-expression of drug efflux pumps and other clearance systems [7,8,9]. Many drugs also suffer from bioavailability barriers and short half-lives. This requires high doses to be orally or systemically administered, which can lead to off-target toxicities, and critical impairment of the host microbiome that wards against disease recurrence and opportunistic superinfections [10,11]. As a result, the last decade has seen a prioritization of new antimicrobial agents designed to slow evolutionary resistance, paralleled by the development of local delivery technologies to address off-target toxicities and pharmacokinetic liabilities.

Accordingly, antimicrobial drug discovery has begun to diverge from conventional small molecule chemical scaffolds to novel biotherapeutics, including nucleic acids [12], phages [13,14,15], and antimicrobial peptides [16,17,18,19,20]. Although these biologics hold significant clinical potential, particularly against MDR microbes [21,22,23,24], their widespread adoption has been limited by immunogenicity, serum instability and off-target effects. Thus, to realize the full potential of these novel antimicrobials as targeted therapies requires local delivery devices that can direct the agent to the site of infection. Antimicrobial supramolecular peptide assemblies have the potential to satisfy both criteria, offering a platform to design fast-acting and potent microbicides that can be engineered for local and specific delivery.

The discovery, design and mechanisms of antimicrobial peptides (AMPs) have been extensively examined elsewhere [25,26,27,28,29,30,31]. Instead, this review will cover assemblies of functional peptide building blocks that exploit their diverse intrinsic and extrinsic antimicrobial effects to develop new supramolecular therapeutic scaffolds. These hierarchical materials are defined by a number of unique properties that are difficult to recapitulate with more traditional platforms, like polymers. For example, the twenty natural amino acids provide an enormous sequence space to explore, with additional complexity offered by an ever-increasing commercial array of non-natural residues. As a result of this chemical versatility, peptide assembly can be driven by electrostatic complexation, hydrophobic interactions or van der Waals forces [32,33], thus allowing for highly modular and customized molecular architectures. Peptides also offer a means to create materials that are inherently bioresorbable and bioactive, often serving as ligands for precise targeting to extracellular protein domains. Finally, the advent of new synthetic techniques and microwave-assisted methods allows peptides to be manufactured at clinically and industrially relevant scales [34,35,36]. Still, membrane-active polymers offer marginal advantages such as the facile and scalable synthesis of high molecular weight material precursors, variable blocks to diversify functionality, and high-persistence length chains that can direct higher ordered entanglement. Nonetheless, the unique advantages of peptides manifest themselves into a class of novel materials that have changed the outlook of clinical antimicrobials and their applications.

**Figure 1:**
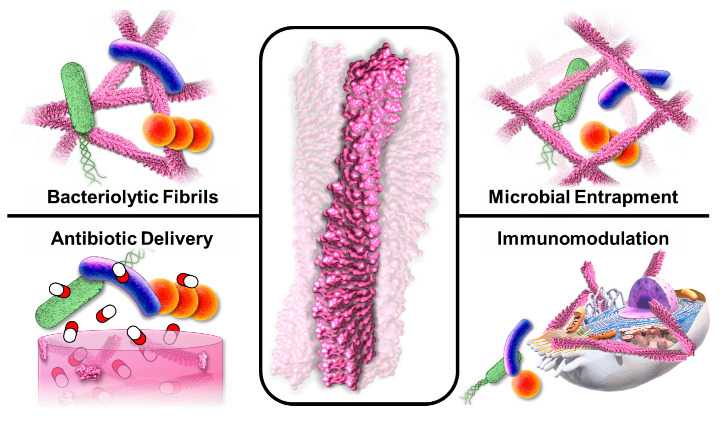
Peptide assemblies adopt diverse supramolecular morphologies to elicit antimicrobial responses via contact-dependent bacteriolysis, delivery of small molecule antibiotics from macroscopic materials, physical entrapment of pathogens in nanonets, or binding to hosts cells to stimulate innate and adaptive immune responses.

Here, we examine peptide-based supramolecular antimicrobials that either serve as the bioactive bactericidal agent, or function as a scaffold for the encapsulation of exogenous antibacterials to improve their specificity, efficacy, and pharmacologic properties (Figure 1). We limit our discussion to non-covalent, self-assembled structures, as biomaterials prepared from chemically ligated peptide conjugates have already been extensively reviewed [37,38,39]. Together, these supramolecular technologies allow clinicians to directly implant or inject the therapeutic material at the infection site, promote passive and active targeting of circulating therapies, and the utilization of non-conventional delivery methods for local application (e.g., aerosolization). In part, such precision over material localization itself alleviates many barriers of bioavailability. Further, biomolecular assemblies consisting of, encapsulating, or bound to, antimicrobials can achieve high local concentrations of the effective agent and provide protection from degradation and clearance, therefore leading to improved efficacy in vivo. More importantly, by exploiting antimicrobials with unique modes of action, or through the revitalization of novel antibiotic candidates that have previously been discarded, these materials can offer unique opportunities to design therapies capable of circumventing MDR. Thus, antimicrobial peptide-based materials represent an emerging weapon in our clinical arsenal against bacterial infections.

## 2. Antimicrobial Supramolecular Peptide Assemblies

AMPs represent a class of naturally derived antimicrobials that have evolved as key defensive systems across multiple ecologic phylum [40,41,42,43]. These bactericides elicit their effects via rapid, often lytic, mechanisms of action that do not principally rely on biochemical targets [44,45,46,47]. Importantly, these membrane-specific physical mechanisms are not shared by conventional antibiotics, and so AMPs are generally active towards MDR microbes. Yet, despite their therapeutic potential, AMPs have not been widely adopted into clinical practice due to several physiological and pharmacological barriers, including proteolytic instability, high serum binding, poor mucosal partitioning and broad-spectrum toxicity [48,49,50]. Organization of these bioactive compounds into higher-ordered, self-assembled structures has the potential to circumvent many of these limitations, while simultaneously adding structural and mechanical functionalities otherwise absent in their monomeric form.

Key to peptide self-assembly are intra- and inter-molecular interactions among hydrophobic, charged, and aromatic residues that drive thermodynamically favorable supramolecular organization. The resultant nanostructures often adopt fibrillar morphologies, which then provide the building blocks for further assemblies to form multimeric nanonets, nanoribbons, nanotubes, and bulk hydrogels. Here, we discuss the antimicrobial activity of nanofiber architectures, as well as their eminent higher-ordered structures, and highlight how supramolecular organization alters their specificity, potency, and clinical relevance.

### 2.1. Monomorphic Nanofibers

Many naturally derived antimicrobial nanofibers adopt amyloid-like architectures, reminiscent of those found in neurologic pathologies [51,52,53,54]. Although β-amyloid nanofibers are the most predominant structures reported in the literature, there exist other conformational states that can achieve similar antimicrobial activity. We focus on systems with a singular morphologic state (monomorphic) and use their divergent structural characteristics to distinguish β-amyloid from non-amyloid fibers, linking molecular conformation to antimicrobial activity.

#### 2.1.1. β-Amyloid Fibrils

Amyloid-β (Aβ) peptides, well known for their role in Alzheimer’s disease [55,56,57], have been recently shown to exhibit antimicrobial activity against many Gram-positive and Gram-negative microbes, as well as fungal pathogens [58,59,60]. The prevailing evolutionary hypothesis is Aβ emerged as a defense against neurologic infections [58,61,62,63], and subsequent structure-activity studies have shown that its activity is derived from an ability to rapidly adopt oligomeric states that precede to protofibril formation and poration of the bacterial cell wall [64,65]. Bacteriologic studies demonstrate broad spectrum activity of Aβ, with micromolar potency against the Gram-positive microbes *E. faecalis*, *L. monocytogenes*, *S. aureus*, *S. epidermidis*, *S. agalactiae*, and *S. pneumoniae* [61,66]. Propagation of these self-assembled structures can also display long-range effects, including the entrapment of microbes within β-amyloid fibrillar arrays to prevent their access to mammalian cells [67]. This suggests that, in addition to their intrinsic bacteriolytic activity, supramolecular structures can inhibit infections by serving as a physical barrier. This is supported by biophysical studies of Aβ assembly in the presence of Gram-negative microbes, including *C. pneumonia*, *T. palladium*, *B. burgdoferi*, and *P. gingivalis*, which were shown to influence pathway-dependent Aβ assembly and increase the production and deposition of β-amyloid fibers [66]. Notably, in vivo studies using a transgenic mouse model of Alzheimer’s Disease (5XFAD) infected with *S. typhimurium* found a greater abundance of Aβ assemblies formed directly surrounding the pathogenic bacteria [68]. By reinforcing the protective properties of these assemblies, *S. enterica* infection of transgenic mice engineered to produce a mutant human isoform of the amyloid precursor protein exhibited a longer survival rate compared to the control group without Aβ expression [69].

**Figure 2:**
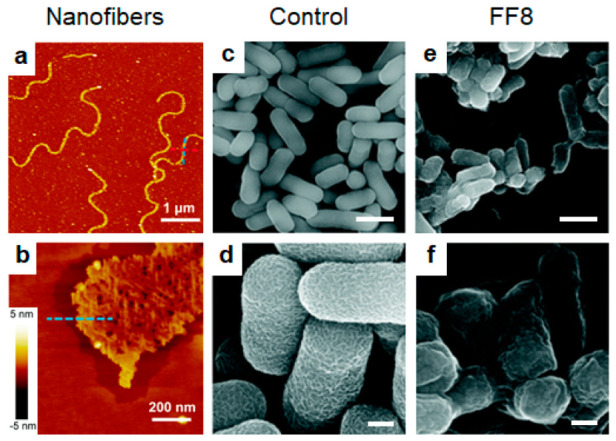
FF8 self-assembly into nanofibers and antimicrobial activity. (**a**) AFM nanofiber assembly with (**b**) zoomed in profile of the edge both observations in a basic environment. (**c**) SEM of untreated *E. coli* with (**d**) zoomed in morphology micrographs. (**e**) SEM of *E. coli* treated with FF8 with (**f**) zoomed in morphology micrographs. Scale bars a, c, e = 1 μm and b, d, f = 200 nm. Adapted from [73] with permission from The Royal Society of Chemistry.

The assembly phenomena of Aβ peptides have been studied for decades, and identified a conserved Phe-Phe (FF) dipeptide motif that drives their organization into higher-ordered states [70]. Intermolecular hydrogen bonding and *π-π* stacking of these residues serves to reduce the self-assembly energy barrier and stabilize resultant nanostructures [71,72]. This has been subsequently exploited to develop synthetic, amyloid-like antimicrobials incorporating FF self-assembly domains. For example, Shen et al. prepared a mirrored 8-mer peptide, KRRFFRRK (FF8), in which the FF core is flanked by basic lysine and arginine residues [73]. FF8 formed stable, micron-length fibers in basic environments (Figure 2a,b), whereas the control peptide KRRGGRRK (GG8) exhibited no higher ordered structure, confirming the importance of the FF core on assembly. Consequently, GG8 was inactive towards *E. coli* cells, while the bulky aromatic groups of FF8 helped to stabilize its intercalation into the lipid outer microbial membrane to elicit bacteriolysis (Figure 2c–f). Interestingly, although FF8 was active toward the tested Gram-negative pathogens, it was relatively inert towards the Gram-positive microbe *S. aureus*. This is explained by the ability of the thick peptidoglycan coat of Gram-positive bacteria to insulate against the lytic effects of the peptide.

Other bulky and hydrophobic residues, such as tryptophan, have also been used to stabilize nanofiber assemblies. For example, the tryptophan-rich, *de novo* peptide MAD1 undergoes tryptophan zippering to form antitubercular structures [74]. Here, intermolecular peptide organization is driven via Trp-Trp pairing, which has been shown to improve the stability and enhance the antimicrobial effects of self-assembled β-hairpin sequences [75]. Interestingly, this tryptophan-mediated assembly imparts MAD1 with high specificity towards *M. tuberculosis*, achieving an MIC of 2.5 μM towards the pathogen with minimal activity toward an array of Gram-positive and Gram-negative microbes.

While these non-natural, linear sequences can be engineered to attack bacterial pathogens, nature appears to prefer cyclic peptide building blocks to construct supramolecular antimicrobials. Exemplifying this are polyphemusin-1 (PM-1) [76,77,78,79] found in horseshoe crab hemocytes, θ-defensin BTD-2 [79,80,81] isolated from baboons and protegrin-1 and -4 (PG-1, -4) [78,79,82,83,84,85] derived from porcine leukocytes, which all adopt cyclic conformations and β-sheet rich amyloid assemblies that potentiate their antimicrobial activity. PM-1, for instance, displays nanomolar activity towards *E. coli* (MIC = 30 nM), with broad spectrum effects towards a multitude of other pathogens [76,77,78,79]. Recent structure-activity studies have linked the β-hairpin secondary structure of PM-1 to its ability to form long-range fibrillar assemblies that mediate receptor-independent mechanisms of bactericidal action [79]. Antimicrobial BTD-2 peptides, which are cyclized via central disulfide linkages, have also been recently observed to self-assemble into anti-parallel oligomers to form asymmetric amyloid-like fibrils [79,80]. Interestingly, the co-assembly of BTD-2 with other fibril-forming sequences, such as the tau-derived AcPHF6 peptide, has been shown to enhance the growth and stability of amyloidogenic assemblies [81]. This suggests that the combinatorial assembly of diverse amyloid-forming peptides could access more structurally stable and potent antimicrobial states compared to homogenous systems. Similarly, protegrin peptides PG-1 and PG-4 display conformationally-dependent, broad-spectrum activity [78,79,82,83], which is potentiated upon oligomerization into β-sheet rich amyloid assembles [84,85]. To demonstrate this, Guor et al. tested the activity of monomeric and self-assembled PG-4 against *B. subtilis* to elucidate gain- or loss- of function after higher order assembly [84]. While the antibacterial potency of PG-4 was similar in the unassembled vs. assembled state, biocompatibility of the supramolecular scaffold towards mammalian cells was significantly improved compared to the monomer, as demonstrated by a reduction in peptide cytotoxicity toward both HEK-293 and Caco-2 cells after 24 h of treatment.

**Figure 3:**
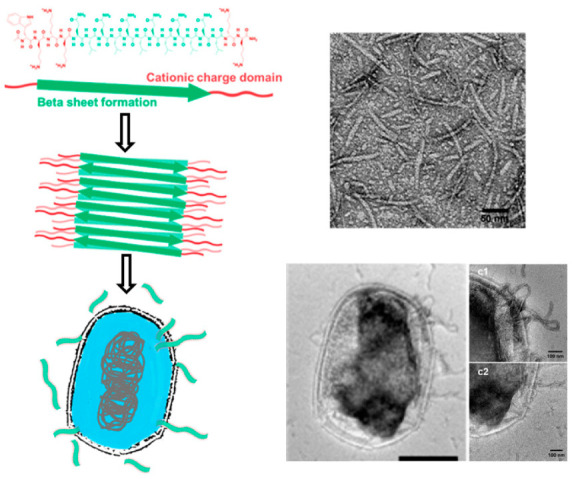
(Left) MDP D-W362 self-assembly into SAANs to form β-amyloid nanofibers. (Top right) Negatively stained TEM of D-W362 forming β-amyloid nanofibers and (bottom right) TEM of E. coli treated with D-W362 (c1 and c2) zoomed into bacterial membrane. Adapted with permission from [86]. Copyright 2018 American Chemical Society.

This behavior of PG-4 serves to highlight one of the principle advantages of supramolecular AMP scaffolds, which is that their microbial specificity and host tissue biocompatibility often significantly improve following assembly. This phenomenon has been exploited to develop self-assembling antimicrobial nanofibers (SAANs) composed of QL dipeptide repeats that are flanked by basic residues, referred to as Synthetic Multidomain Peptides (MDP) (Figure 3) [86,87]. Here, hydrogen bonding and hydrophobic intermolecular interactions between the QL central motif promotes the formation of β-amyloid fibers, which then interact with and destabilize bacterial cell membranes. The addition of lysine residues on the termini enables modulation of fiber length, strength, entanglement, and gelation through the adjustment of pH and salinity [88]. More recent studies have focused on tuning the antimicrobial activity of SAANs by either conjugating the sequence to an AMP [89], or optimizing the sequence composition to enhance its intrinsic antibacterial specificity [86,87,90]. For example, conjugation of melittin, a cytotoxic AMP, to a histidine-containing MDP (H_3_(QL)_6_-Mel) resulted in a dramatic decrease in its cytotoxicity towards mammalian cells, without impacting its antimicrobial potency [89]. This was a result of sequestering the hydrophobic melittin residues within the β-amyloid nanofiber core to reduce its interaction with lipid mammalian cell membranes, while simultaneously presenting the antimicrobial warhead of the peptide orthogonal to the fibril axis to maintain its bactericidal activity. As an alternative strategy, Xu et al. found that the tryptophan- and lysine-rich MDP, D-W362 (^d^W^d^K_3_(QL)_6_^d^K_2_), formed fibers with potent antibacterial activity and minimal cytotoxicity (Figure 3) [87]. The optimized sequence, which contains a mix of L and D amino acids, was found to be toxic towards *E. coli*, *P. aeruginosa*, *S. aureus*, and *S. epidermidis* with MIC values ranging from 5–20 μM. Counter-screening the sequence against bone marrow-derived mouse monocytes revealed a conformationally dependent toxicity profile. For instance, at 1 μM concentrations, where MDPs exist as monomers or small oligomers, ~50% cytotoxicity of healthy cells was observed following a 24 h incubation. However, when MDP concentration was increased to induce self-assembly (>5 μM), no appreciable cell death was observed. Biophysical structure-activity studies using model liposomal membranes and whole *E. coli* bacteria showed that β-amyloidogenic assemblies of D-W362 were able to deform and rupture the microbial membrane, but prevented accumulation of the peptide in mammalian cell or erythrocytes membranes to avoid cytotoxicity [86]. The design of these antimicrobial assemblies has been more recently expanded to include poly-histidine blocks (WH*_X_*(QL)_6_K_2_ where *x* = 5, 7, or 9) in order to promote their pH-dependent assembly within acidic macrophage lysosomes that serve as a survival niche for certain bacterial pathogens, such as *B. fragilis* [90]. These sequences maintain their β-amyloid fibrillar state at neutral pH, but undergo instructed disassembly in acidic environments due to histidine protonation and electrostatic repulsion, yielding bioactive monomers that can elicit bacterial lysis. When tested in aerobic settings at neutral pH, the peptide was well tolerated by the model microbe *E. coli* (MIC > 40 μM). However, under anaerobic conditions, where the culture media became acidified to pH 6.4, the peptide potently killed *E. coli*, *B. fragilis* and *S. aureus* pathogens with MICs of 10, 5, and 5 μM, respectively. As designed, the polyhistidine sequence maintained is biocompatible properties and was well tolerated by NIH/3T3 fibroblasts (>80% viability up to 40 µM) and red blood cells (<5% hemolysis observed at concentrations 16 times greater than their bacterial MIC).

#### 2.1.2. Non β-Amyloid Fibril Assemblies

Although β-amyloid antimicrobials have been most widely studied in the literature, several helical supramolecular bactericides have also been described, which is perhaps unsurprising given the well-established bacteriolytic activity of AMP α-helices. In two notable examples, helical antimicrobials were derived from peptides in the phenol-soluble modulin (PSMs) family, PSMα3 [91,92]. This peptide is a key virulence factor of *S. aureus* and, once secreted, is toxic toward mammalian cells. However, the α-helical PSMα3 monomer is inactive toward host cells, and only displays its cytotoxic effects after oligomerization of the peptide to form cross-α fibrillary architectures. Investigation of this sequence identified the amphipathic LFKFFK functional motif responsible for its supramolecular assembly [93]. Surprisingly, this peptide exhibited strong dose-dependent antibacterial activity against *M. luteus* and *S. hominis* suggesting that, in addition to its ability to elicit host toxicity, PSMα3 may serve as a defensive agent of *S. aureus* to ward off competing microorganisms. Later, the self-assembled structure of the six residue sequence was found to exist as two specific polymorphic conformations: an atypical β-sheet hexamer that forms a supramolecular assembled nanotube and non-perpendicular out-of-register β-sheets forming cross-β fibrils. These divergent assembly phenomena suggest a structural plasticity of the supramolecular architecture that generates various virulent outcomes dependent on the local microenvironment. Similarly, two other members of the same peptide family, PSMα1 and PSMα4, adopt cross-β conformations leading to a class 1 steric zipper in β-amyloid-like assemblies [93]. Segmental analysis identified the minimal self-assembly motifs IIKVIK and IIKIIK, for PMSα1 and PMSα4, respectively, that drive self-assembly. These assembly domains on their own did not show functional antimicrobial activity, supporting the assertion that fibrillization itself does not confer activity but rather enhances the bacteriolytic effects and specificity of antimicrobial motifs. Other antimicrobial sequences derived from the PSM family of peptides have also been recently engineered to combat MDR bacteria [94]. The lead candidate peptide zp3 (GIIAGIIIKIKK-NH_2_) displayed an MIC of 8 μM toward *E. coli*, *B. subtilis*, and *C. freundii*, and generated minimal hemolytic activity (<5% at 256 μM) and cytotoxicity toward HEK293 cells (IC_50_ > 80 μM) when employed well above its microbicidal concentration.

**Figure 4:**
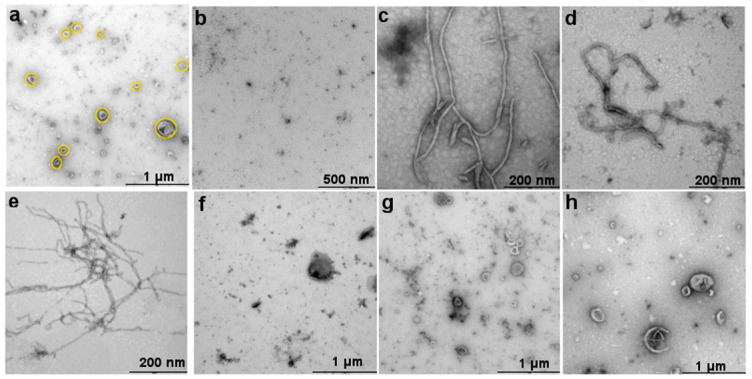
(**a**) TEM images of liposomes before peptide injection (yellow circles); (**b**) LL-37 alone; the effect of 10 μΜ LL-37 on (**c**) neat DMPC; (**d**) DMPC:DMPG (4:1); (**e**) DMPC:DMPG (3:2); (**f**) DMPC:cholesterol; (**g**) DOPC; and (**h**) DOPC:DOPG (4:1). Image adapted from [99] under the Creative Commons Attribution 4.0 International License http://creativecommons.org/licenses/by/4.0/.

Other examples of AMP assembly resulting in amplified activity can be found in the literature, such as the α-helical cathelicidin (LL-37) [95]. Here, peptide monomers organize into an anti-parallel dimer which enables a broader spectrum of microbial toxicity compared to its monomeric form. This is a result of the altered orientation of bulky hydrophobic residues following dimerization, which promotes dimer intercalation into cell membranes to induce toxicity. Subsequent long-range assembly of LL-37 in the presence of lipid membranes leads to the formation of helical fibers (Figure 4). These fibers are able to extract membrane lipids from bacteria and sequester them within hydrophobic pockets in the dimerized fibrils to prevent their reintegration into the microbial membrane [96]. These membrane-templated assemblies have also been visualized for the α-helical antimicrobial peptide Lasioglossin-III, which forms protofibrillar structures on mammalian cell membranes following fusion [97]. Similarly, early studies hypothesized conformational rearrangement of LL-37 from α-helix to β-structures promoted its assembly into amyloidogenic architectures [98]; however, this was refuted after β-structures were not observed following peptide assembly in lipid membranes [96,99]. This conclusion was reinforced when the co-assembly of LL-37 with Aβ peptides not only inhibited Aβ fibril growth, but destabilized pre-formed assemblies by inhibiting β-sheet secondary structure [100].

### 2.2. Nanofibrillar Nets

**Figure 5:**
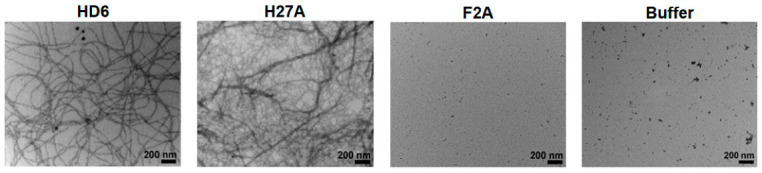
TEM micrographs of wild type HD6 and synthesized amino acid mutant nanonet formations in buffer. Scale bar = 200 nm. Adapted with permission from [101]. Copyright 2014 American Chemical Society.

Long-range propagation of amyloid fibrils can yield antibacterial nanonets, where the evolution of fibrillar meshes physically traps nearby bacteria to elicit antimicrobial effects by preventing microbial invasion, rather than direct killing of the invader. While Aβ has been reported to entrap pathogens, its primary mode of antimicrobial activity is lysis of the sequestered bacteria [67,68]. Human α-defensin 6 (HD6), on the other hand, has been well characterized to self-assemble into supramolecular nanonets that elicit their function through bacterial entrapment (Figure 5) [101,102,103,104]. This peptide, naturally secreted by Paneth cells in the human gastrointestinal tract, serves to limit tissue invasion by pathogens and pathobionts in the gut [102,103]. Self-assembly of peptide monomers, driven largely by hydrophobic and aromatic interactions, leads to the formation of β-amyloid nanofibers that are ~80 nm in width [104], and merge with neighboring fibrils to form net-like structures upon interaction with external flagella and fimbrae of bacteria [105]. Stabilization of the resultant nanonets occurs after disulfide linkage of regiospecific cysteine residues to form the covalent array [101,102]. Fascinatingly, fibril morphology can be readily tuned by changing the identity of hydrophobic and aromatic residues. For example, His27Ala (H27A) and His27Trp modifications result in robust fibrillization, while Phe2Ala (F2A) and Phe29Ala mutations inhibit self-assembly and instead result in the formation of amorphous aggregates (Figure 5) [101]. Nanonets prepared from such sequences inhibit the invasion of *S. enterica* and *L. monocytogenes* gastrointestinal pathogens in vitro [101], and are cross-reactive with the pathogenic yeast *C. albicans* [102]. Co-culture experiments demonstrated the selective capture of *S. typhimurium* pathogens mixed with T84 epithelial cells by HD6, resulting in a reduction in bacterial invasion comparable to the minimally invasive *InvA* bacterial mutant [106]. This was confirmed in vivo with HD6 transgenic mice to find that there was a reduction in *S. typhimurium* numbers in internal organs where HD6 was expressed, with no change in total numbers in the intestinal lumen, confirming the entrapment propensity of HD6 to prevent invasion. The nets continue to trap the bacterial pathogens through the lumen until they can either be excreted or attacked by the host immune system.

Multiple other peptides, such as HD5 [107] of the α-defensin family and hBD1 [108] of the β-defensin family, also trap and kill pathogens such as *E. coli*, *S. aureus*, and *K. pneumoniae*. HD5 undergoes a proteolytic activation to generate a minimal sequence with broad and potent activity [107], while the protease-activated form of hBD1 assembles to create nanonets that are proteolytically stable and prevent microbe migration [108]. Big defensins, primordial ancestors to β-defensins that are produced in marine organisms, have also been reported to exhibit antimicrobial effects through the formation of fibrillar nanonets [109,110,111]. Conserved domains among the peptides in this class include a hydrophobic N-terminal motif that promotes amyloidogenic assembly, and a cationic C-terminal block containing three cysteine residues that collectively act to electrostatically bind pathogens and seal the net via disulfide cross-linking, respectively. Exemplifying the functional activity of these net-forming peptides is *Cg*-BigDef1, produced by the oyster *Crassostrea gigas* [109]. *Cg*-BigDef1 shows broad-spectrum activity towards a variety of Gram-positive and Gram-negative microbes, and notably exhibited acute growth inhibition of both drug-sensitive and multidrug-resistant strains of *S. aureus* (MIC = 1.25–5 μM). Mechanistic bacteriologic studies demonstrate that *Cg*-BigDef1 acts by forming a fibrillar net that tightly adheres to the nearby bacteria. Highlighting their clinical potential, the treatment of mammalian NCI-H292 bronchial epithelial cells with *Cg*-BigDef1 did not cause observable toxicity, as assayed via release of lactate dehydrogenase [109]. Similarly, *Ap*BD1, a nano-net forming big defensin produced by the scallop *Argopecten purpuratus*, exhibited antimicrobial activity towards *S. aureus* [110,111]. Mutagenesis studies on *Ap*BD1 demonstrated the specific importance of the conserved hydrophobic N-terminal in driving nanonet assembly of big defensins, which was profound when the C-terminal cysteine-rich domains were disrupted upon cysteine to arginine mutation not impacting structural formation [109].

### 2.3. Nanoribbons

**Figure 6:**
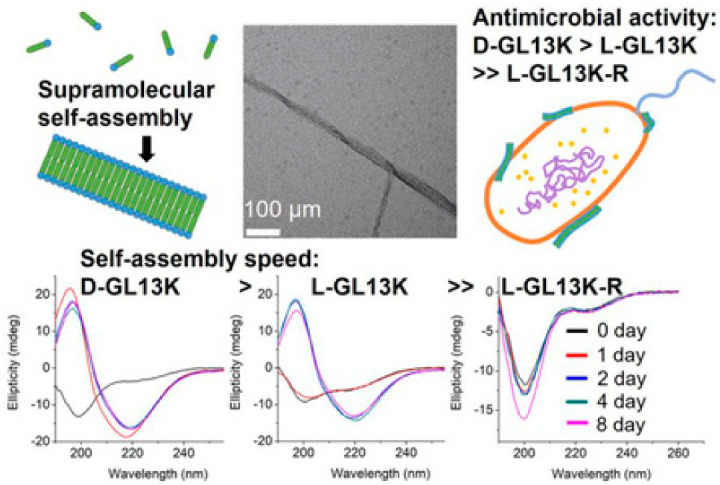
(Top) L-GL13K nanoribbon self-assembly schematic, SEM image of GL13K nanoribbon, and cartoon of bacterial membrane interaction (from left to right). (Bottom) Circular dichroism spectra of D-, L-, and scrambled GL13K analogues as a function of time. Reproduced from [118] with permission from The Royal Society of Chemistry.

The ability to tune the molecular, nanoscale, and microscopic topology of peptide-based nanostructures has advanced considerably over the last two decades. It is now well established that altering the super-secondary structural state of short, amphiphilic peptides can yield architectures that diverge from traditional fibrils, including flat and twisted nanoribbons. These 2D nanostructures can emerge independently in solution, or be triggered in the presence of anionic surfactants (e.g., SDS) [112], amphiphilic dyes (e.g., indocyanine green) [113], and Fmoc-modified amino acids [114]. While these materials often serve as platforms to study hierarchical assembly phenomena, their emergent and tunable antimicrobial properties have also recently garnered interest. GL13K for example, a peptide engineered from a human salivary protein, self-assembles to form nanoribbons (Figure 6, top) that exhibit potent antimicrobial activity at single digit μg/mL concentrations towards *P. aeruginosa* and *E. coli* [115]. Biophysical studies in model bacterial liposomal membranes demonstrate that the peptide undergoes multiple conformational changes during lipid insertion-adopting random coil in solution, α-helical at low lipid concentrations, and finally β-sheet in liposome-rich solutions [116]. These mechanisms are specific to microbial membranes, resulting in low levels of off-target hemolysis in vitro (~5%) and clinically relevant safety profiles in vivo [115]. Interestingly, D-enantiomeric GL13K sequences show a marked improvement in potency relative to their natural L-counterparts, which cannot be entirely explained by enhanced proteolytic stability. For example, Hirt and co-workers demonstrated that D-GL13K has superior efficacy compared to L-GL13K in both wild-type *E. faecalis*, and a protease-deficient strain of the same pathogen [117]. This suggests protease resistance is not the only mechanism favoring the D-enantiomer peptide. To test this assertion, circular dichroism and cryo-TEM experiments were performed to study the temporal changes in L- and D-GL13K structure [118]. They found that, although the higher ordered structural states were similar between the enantiomers, the rate of nanoribbon formation is accelerated for D-GL13K compared to its L- analogue (Figure 6, bottom).

Interpretation of this phenomenon requires a systematic understanding of the structure-activity relationship defining nanoribbon formation. One key parameter is the identity of terminal residues on di-phenylalanine based peptide amphiphiles, which has a profound effect on their ability to assemble into nanoribbons. Using three phenylalanine rich peptides (EFFFFE, KFFFFK, and EFFFFK) to monitor β-sheet stacking, Hu et al. found that electrostatic repulsion between termini was responsible for twisting β-sheet tapes into ribbons [72]. Conversely, electrostatic attractions of oppositely charged termini led to the lamination of β-sheets into belt like structures. Although these sequences were not tested for antimicrobial properties, the fundamental understandings obtained through the study provides design criteria for future engineered antibacterial nanoribbon assemblies.

### 2.4. Nanotubes

**Figure 7:**
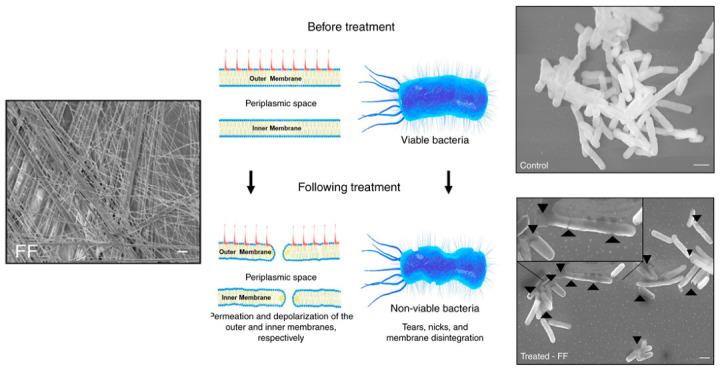
Diphenylalanine self-assembly and antimicrobial activity. (Left) SEM image of self-assembled nanofibers. Scale bar = 10 μm. (Center) Schematic of proposed antimicrobial mechanism via membrane disruption and permeation. (Right) SEM of *E. coli* treated with GG (control) or FF nanotubes. Arrows mark altered membrane morphology caused by nanotube interactions. Scale bar = 1 μm. Image adapted from [71] under the Creative Commons Attribution 4.0 International License http://creativecommons.org/licenses/by/4.0/.

The supramolecular diversity of FF-containing peptides has also enabled the fabrication of hollow fibrils, or nanotubes, constructed through the gradual annealing of heated peptide solutions (Figure 7) [71]. When added to bacterial cultures, diphenylalanine showed antimicrobial activity as potent as 125 μg/mL against *E. coli* and *L. monocytogenes*, and 250 μg/mL toward *R. radiobacter* and *S. epidermidis*. Mechanistic studies using complementary membrane probes demonstrated that the peptide was not only able to disrupt the integrity of the outer microbial membrane, but also disrupted the inner membrane to yield complete membrane depolarization and bacterial lysis. Similar to previous investigations, FF nanotubes were found to be generally biocompatible against healthy cells and erythrocytes after an overnight incubation.

**Figure 8:**
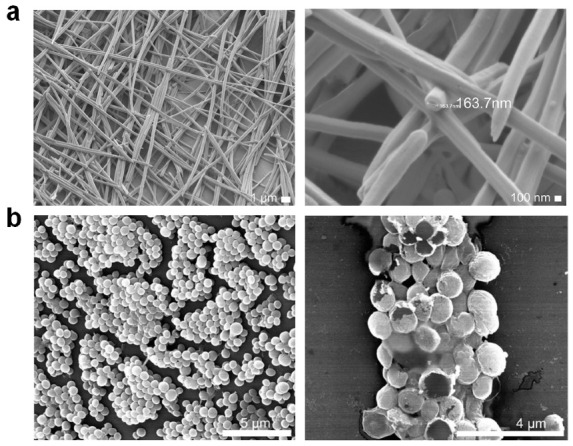
SEM of NH_2_-FF-COOH (**a**) nanotube self-assembly and (**b**) *S. aureus* treated with 2.5 mg/mL peptide. Adapted from [119] with permission from Elsevier.

Follow up studies to elucidate the mechanism of nanotube assembly and activity against bacterial biofilms employed three FF dipeptides with modified end termini and residue chirality (NH_2_-FF-COOH, NH_2_-ff-COOH, NH_2_-FF-NH_2_, lowercase designates D-amino acids) (Figure 8) [119]. Both dipeptides with C-terminal carboxylates showed preferential activity towards *S. aureus* derived lipid membranes, and were capable of eliminating *S. aureus* biofilms at 10 mg/mL. This activity was suggested to be the result of two complementary mechanisms, where the surfactant-like dipeptide disrupts the *S. aureus* biofilm matrix, and the interpolation of FF into bacterial membranes leads to ion channel formation and membrane depolarization. Interestingly, the dipeptides retained their membrane disruptive potential in *E. coli*, but were unable to inhibit biofilm formation by this pathogen. The carboxy-terminated dipeptides were also found to be non-cytotoxic and hemocompatibile up to 10 mg/mL, while the NH_2_-FF-NH_2_ analogue resulted in 80% cell death at 2.5 mg/mL. It is suggested that the increased formal charge of the N-terminal amidated dipeptide is responsible for its cytotoxicity.

### 2.5. Macroscopic Fibril Gels

**Figure 9:**
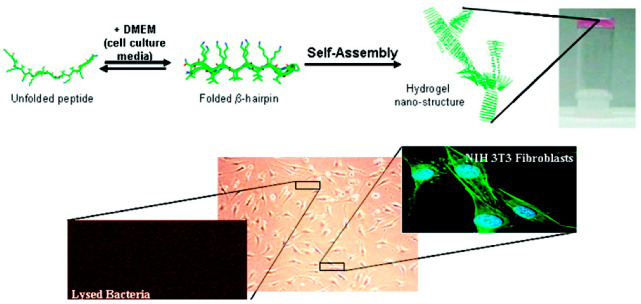
MAX1 hydrogelation mechanism and targeted antibacterial activity in co-culture. Adapted with permission from [124]. Copyright 2007 American Chemical Society.

Peptide nanofibers can propagate in solution to form macroscopic antimicrobial hydrogels. This offers the development of injectable microbicidal materials where the fibers serve as both the scaffolding material and the antibacterial agent [120,121,122]. As a demonstration of this, the Schneider group has developed a suite of β-hairpin self-assembling antimicrobial peptides, including PEP6R [123], MAX1 [124,125,126,127], and MARG [128], composed of two arms of alternating valine and cationic residues that flank a turn-promoting tetrapeptide sequence (-V^D^PPT-). The hexa-arginine PEP6R peptide exhibits potent growth inhibition of various Gram-positive and Gram-negative pathogens at as little as 0.5 wt%, with minimal off-target cytotoxicity and hemolysis [123]. Mechanistic studies reveal that these gels destabilize bacterial envelopes via the formation of hydrogen bonds and salt bridges between the guanidinium groups of the peptide and phosphate-rich head of bacterial membrane lipids and glycoproteins. Structure-activity relationships using sequence analogues containing 2–8 arginines’ showed that all peptides within this family elicit potent growth inhibition of *S. aureus* and *E. coli*, with sequences containing ≥4 arginine residues showing a greater specificity for *E. coli*. Additionally, the elastic moduli of the materials was found to increase with greater arginine content of up to six residues. Finally, the lead peptide gelator, PEP6R, was able to completely inhibit the growth of drug-resistant *P. aeruginosa* at 1.5 wt%, and displayed shear-thinning and self-healing properties after syringe delivery [129], suggesting its clinical potential as an injectable tissue filler to prevent post-surgery infections. Similarly, the MAX1 peptide, which is compositionally analogous to PEP6R other than the substitution of arginine residues with lysine [126], exhibits broad spectrum activity towards the Gram-positive pathogens *S. epidermidis*, *S. aureus*, and *S. pyogenes*, as well as the Gram-negative microbes *K. pneumoniae* and *E. coli,* at 2 wt% [124]. Importantly, MAX1 gels were able to preferentially kill the Gram-negative pathogens *Achromobacter xylosoxidans* and *Stenotrophomo maltophilia* in a co-culture experiment with NIH 3T3 fibroblasts (Figure 9) [125], without significant off-target cytotoxicity to the mammalian cells or erythrocytes [124]. Blending these designs, the MARG1 peptide was engineered with two lysine → arginine substitutions in the parent MAX1 sequence to create a shear-thinning, injectable hydrogel with optimized mechanical properties and bactericidal efficacy against methicillin-resistant *S. aureus* [128].

Structure-activity studies of other β-sheet rich fibrillar hydrogels prepared from multidomain peptides (MDPs) have begun to identify the role of supramolecular assembly on the antimicrobial effects of such materials. For example, K_3_W(QL)_6_K_2_ was found to exhibit the most potent antimicrobial activity over other compositional analogues, including WK_2_(QL)_6_K_2_ and K_2_W(QL)_6_K_2_, due to its decreased potential for multimeric oligomerization (MIC = 20, 160, >160 μM, respectively) [87]. This reduced self-assembly propensity allowed the K_3_W(QL)_6_K_2_ peptide monomer to more readily interact with bacterial membranes and cause cell lysis. When formed into hydrogels, however, the opposite phenomenon was observed, where the more tightly packed nanofibers seen with WK_2_(QL)_6_K_2_ and K_2_W(QL)_6_K_2_ exhibited greater growth inhibition of *S. aureus* (74% and 92% inhibition, respectively, at 2 wt%) [130]. Here, greater entanglement of the fibrillar hydrogel mesh was able to more efficiently trap bacteria within the matrix to inhibit proliferation via lysine-lipid interactions. Similar lysine-dependent growth inhibition has been observed for hydrogels prepared from the amphipathic surfactant like peptide A_9_K_2_, with growth inhibition demonstrated towards *S. aureus*, *B. subtilis*, *E. coli* and *P. aeruginosa* [131].

While activity against planktonic cells is important, the inhibition of multicellular bacterial biofilms is a particularly attractive goal for clinical biomaterials. Kumar and colleagues described a series of cationic and amphiphilic self-assembling, β-sheet forming peptides (CASP) that contain 2–8 lysine residues in their sequence to form hydrogels that could inhibit the formation of *P. aeruginosa* biolfims [132]. These sequences tolerated up to six lysine residues, with CASP-K8 not able to form gels at 20 mg/mL concentrations, leading to the team further validating the anti-biofilm activity of CASP-K6. In other work, C-terminal addition of di-lysine or di-orntithine onto Fmoc-diphenylalanine was studied for its ability to prevent the growth of established *S. aureus, S. epidermidis*, *P. aeruginosa*, and *E. coli* biofilms [133]. While di-phenylalanine hydrogelators have been the most widely studied [134,135,136], other dipeptide combinations of Fmoc-FY, -YS, and -YN have been reported to form hydrogels that exhibit antimicrobial activity toward *E. coli* [137]. Interestingly, these tyrosine containing amphiphiles are designed to be enzymatically activated by alkaline phosphatase, found in the periplasmic space in *E. coli*, to potentiate their selective assembly within the pathogen cell envelope. Finally, even the minimal Fmoc-Phe [138] and Fmoc-Trp [139] gelators have been reported to exhibit antibacterial activity against *S. aureus* following self-assembly.

## 3. Combinatorial Strategies

While pure, peptide-based supramolecular antimicrobials have attracted significant attention in their own right, an equally popular strategy is to blend bioactive sequences with other bactericidal or bacteriostatic agents to develop combinatorial materials. Alternatively, the peptide itself may not elicit an antimicrobial response on its own, but rather act as an excipient to enhance the pharmacologic properties of an active agent. In this section, we collectively refer to these combinations as “hybrid co-assemblies”. Potential options for such elements once again represent a diverse collection of molecules, ranging from small molecule antibiotics to nucleic acids, and others. Importantly, while it is well established that bacteriolytic peptides can facilitate the activity of other agents, the antithetical design, in which the co-assembled agent instead facilitates activity of the peptide, must also be recognized.

### 3.1. AMP-Antimicrobial Hybrid Materials

While the addition of more material and drug components adds to the synthetic and toxicologic complexity, benefits of such strategies often lie in unique biochemical and bioresponsive properties afforded by the design [140]. For example, the co-delivery of peptides with antibiotics often enhances the antimicrobial activity of the small molecule inhibitor [141,142]. More importantly, resistance development is minimized by exploiting a variety of antimicrobial vulnerabilities, a strategy already used in high risk infectious diseases, such as HIV and Tuberculosis.

**Figure 10:**
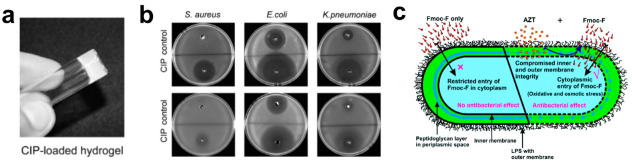
(**a**) ^D^LFF hydrogel loaded with ciprofloxacin (CIP). (**b**) Zone of inhibition experiments demonstrating enhanced antimicrobial activity of CIP-loaded gels (CIP), relative to unloaded gels (control) against multiple bacterial species. Adapted from [147] with permission from Elsevier. (**c**) Mechanistic schematic demonstrating synergistic activity of co-delivered aztreonam and Fmoc-FF. Adapted from [148] with permission from The Royal Society of Chemistry.

Incorporating supramolecular assembly into the design of these systems offers additional advantages of increasing local therapeutic potency, reducing off-target effects and avoiding rapid systemic clearance. For example, the encapsulation of antimicrobials within self-assembled peptide hydrogels enables the formulation of drug depots that sustainably release bactericidal therapies to sites of infection, which is particularly important where biofilms are a concern. Moreover, the antibacterial activity of the peptide hydrogel itself often becomes significantly more potent when paired with an encapsulated antimicrobial agent, often by destabilizing the bacterial envelope to aid in the transmembrane diffusion of the co-delivered compound [143,144,145,146]. As an exemplary application of this strategy, ciprofloxacin, a poorly water soluble antibiotic, was loaded into antimicrobial ^D^LFF peptide hydrogels and tested against various Gram-negative pathogens (Figure 10a,b) [147]. The combination improved the therapeutic activity of the antimicrobial peptide scaffold by 1000- and 500-fold against *E. coli* and *K. pneumoniae*, respectively, while maintaining a similar hemocompatibility (0.9% hemolysis) as the unloaded control matrix. In this scenario, both components benefit from the presence of the other. A similar system encumbered aztreonam and polymyxin B within Fmoc-FF gels [148]. This synergistic system impressively resulted in a 60-fold improvement in component MIC when treated together, and a fractional inhibitory concentration (FIC) of 0.03 against *P. aeruginosa* (Figure 10c). A defining advantage inherent to such an approach is the simplicity with which drug can be encapsulated into the bulk injectable material [149,150,151,152,153]. In each scenario, drug stocks are simply added to the solution of peptide hydrogelator and become trapped in the biopolymeric network after assembly. A unique, yet similar, strategy conjugated a four amino acid, self-assembling motif (FFKK) to nonsteroidal anti-inflammatory drugs (NSAIDs) [154]. Interestingly, naproxen complexes provided significant bacterial inhibition over other NSAIDs, such as ibuprofen and indomethacin. The conjugated peptide sequence provides gelation, affording the drugs improved retention and selective localization.

Metal ions have also seen frequent use in the construction of supramolecular materials through non-covalent coordination with peptides to template their assembly [155]. A common approach is chelation of multivalent cationic species, such as calcium [156,157,158]. Heavy metals, however, also possess additional antimicrobial properties that have been leveraged for biomedical applications. Colloidal gold, for example, is generally bioinert towards eukaryote cells, but is toxic to prokaryotes at sizes <2 nm; encouraging the incorporation of gold-based nanotherapeutics into antimicrobial biomaterials [159,160]. In one example, gold nanodots functionalized with 1-dodecanethiol on their surface enabled the immobilization of the cyclic antimicrobial lipopeptide Glu-Leu-^D^Leu-Val-Asp-^D^Leu-Leu via nonspecific hydrophobic interactions between alkyl chains [159]. Here, 1-dodecanethiol was employed as it has been shown to induce photoluminescence following deposition upon metal nanomaterial surfaces [161,162], which affords subsequent imaging of internalization and localization without compositional alterations or chemical conjugation that may impact biological behavior of the material. This self-assembled nanodot improved the recovery and squamous healing of a methicillin-resistant *S. aureus* (MRSA)-infected in vivo wound model relative to free peptide, with sterilization of the wound observed 6 days after MRSA inoculation in nanodot treated animals [159].

**Figure 11:**
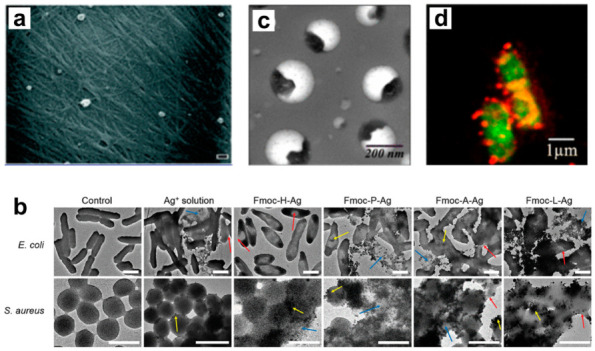
(**a**) SEM images of silver nanoparticles entangled within a napthelene-functionalized tripeptide hydrogel network. Adapted from [163] with permission from The Royal Society of Chemistry. (**b**) Demonstration of membrane fusion (red arrows), clumping (yellow), and disintegration (blue) of various peptide-silver nanoparticle complexes. Image adapted from [164] under the Creative Commons Attribution 4.0 International License http://creativecommons.org/licenses/by/4.0/. (**c**) High-resolution TEM images of zinc-coordinated poly peptide nanogels (PNGs). (**d**) Confocal microscopy of PNG (red)-treated *E. coli* cells (green). Adapted with permission from [165]. Copyright 2019 American Chemical Society.

Like gold, silver nanoparticles also exhibit strong antimicrobial effects that can be improved once incorporated into a self-assembled peptide hydrogel. Gels prepared from a napthelene-functionalized tripeptide ((Nap)Phe-Phe-Cys), and loaded with 80 µg/mL of silver nanoparticles (Figure 11a), completely inhibited MRSA growth and slowed the proliferation of *A. baumannii* by 24 h, while the native gel showed no antimicrobial effects [163]. These same formulations were generally well tolerated by human HeLa cells, maintaining ~90% viability at therapeutically relevant concentrations. Similar work demonstrated significant growth inhibition by silver-loaded Fmoc-amino acid gels [164]. Impressively, this work demonstrated membrane fusion and disruption in both Gram-positive and Gram-negative bacteria, regardless of the amino acid base used for gelation (Figure 11b). Sugar-functionalized polypeptide nanogels, prepared from poly(arginine-r-valine)-mannose, have employed other metal ions, such as zinc, (Zn^2+^), iron (Fe^3+^), and copper (Cu^2+^) to impart antimicrobial properties to the biomaterial assembly [165]. Preliminary screenings of co-assemblies formed from the mannose-conjugated scaffold and metals showed Zn^2+^ as the lead candidate due to antimicrobial activity, mammalian cytocompatibility and assembly proficiency (Figure 11c) [166]. Here, ~100 nm spherical particles were produced and displayed potent antimicrobial activity (MIC = 2 to 16 μg/mL, Figure 11d). Further, coordination of Zn^2+^ to poly(arginine-r-valine)-mannose decreased the peptide’s toxicity towards mammalian fibroblasts and hemolytic potential at concentrations 25 times higher than bacterial MICs, thus widening its therapeutic window.

### 3.2. Peptide-Excipient Co-Formulations

In contrast to the bioactive hybrid assemblies discussed above, many applications exploit bioinert materials to improve the formulation or pharmacologic properties on an active antimicrobial. Remarkably, nearly every class of biomaterial, from synthetic polymeric scaffolds to nucleic acid networks, have been successfully paired with bioactive peptides to augment their antimicrobial toxicity.

**Figure 12:**
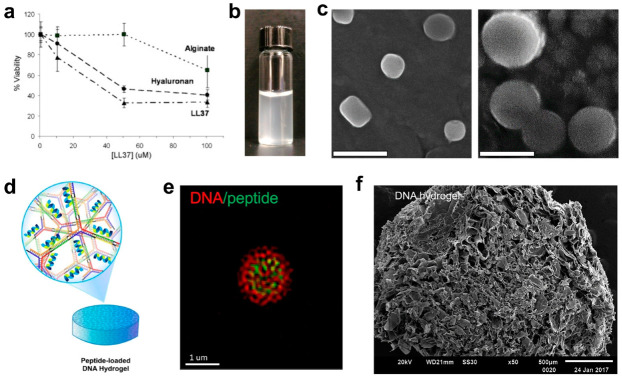
(**a**) Cytocompatibility of LL-37 and LL-37-carbohydrate complexes against human bone cells. Adapted from [169] with permission from Elsevier. (**b**) Solution turbidity of nanogels formed by electrostatic complexation by hyaluronic acid (HA) and poly-L-lysine (PLL). (**c**) SEM images of HA-PLL nanogels in water (left) and following swelling in physiologic medium (right). Scale bar = 500 nm. Adapted from [176] with permission from Elsevier. (**d**) Schematic detailing of Y-scaffolds and peptide loading strategy in antimicrobial hydrogels. (**e**) Confocal microscopy showing localization of nucleic acid matrix and encapsulated peptide. (**f**) SEM image of DNA-peptide matrix. Adapted from [188] with permission from Elsevier.

Perhaps the most common co-assembly involves the use of polysaccharides, such as alginate [167,168,169,170], chitosan [171,172,173], and hyaluronic acid [168,174,175,176]. Lesser known compounds have also been successfully implemented in peptidic assemblies, such as glucomannan and chitin [177,178]. Carbohydrates are popular due to their general cytocompatibility, cell sensing, stability and ease of manufacture. Additionally, many material and biochemical parameters, such as polysaccharide concentration, molecular weight, and enzyme sensitivity, can be manipulated to obtain the desired rate of cargo release [177]. Some AMPs have shown high affinity for polysaccharide excipients, affording compositionally simple yet robust materials. For example, the human defensin LL-37, a natural cathelicidin employed within leukocytic lysosomes [179], binds with high affinity to sodium alginate [169]. While minimizing the cytotoxic effects of LL-37 on MG-63 bone tissue cells, the peptide-polysaccharide system maintains the antimicrobial activity of free LL-37 (Figure 12a). Interestingly, an ideal loading of the peptide appears to maintain an optimal equilibrium of complexed and free peptide. Work by Medina and co-workers electrostatically complexed poly-L-lysine, a polycationic peptide with generally weak antimicrobial activity, to hyaluronic acid to form nano-scale hydrogels, or nanogels [176]. Here, electrostatic assembly of the particle enables various biomolecular cargo present during synthesis to be readily encapsulated within the carrier core (Figure 12b,c). This was shown utilizing vancomycin as the cargo antibiotic, leading to a 4–15 fold increase in drug activity towards a panel of Gram-positive and Gram-negative bacteria. This enhanced efficacy is thought to result from the binding of microbes to the nanogel hyaluronic acid corona, which digest the extracellular carbohydrate as a nutrient source during pathogenesis. Contact-mediated destabilization of the microbial envelope upon interaction with poly-l-lysine in the particle cooperatively enhances the diffusion of vancomycin and access to its ^D^alanyl-^D^alanine target. In a similar fashion, nisin Z, a 34 amino acid polycyclic antimicrobial peptide produced by *Lactococcus lactis*, was conjugated to hyaluronic acid to facilitate supramolecular assembly of the hybrid into antibacterial gels that could be incorporated into wound dressings, contact lenses, and cosmetics formulations [180]. Uniquely, this system was shown to maintain the peptides’ bioactivity both as a solution and crosslinked hydrogel.

An alternative to carbohydrates is the complexation of peptides with nucleic acids to enable hybridization-mediated self-assembly [181,182,183,184]. Nucleic acids also offer several superior modes for material design. Principally, both DNA and RNA can be custom designed from the bottom up to provide complete control over a vast chemical library [185,186]. Such architectures are also logically and chemically analogous to a key building block of non-covalent assemblies in biology: protein–nucleic acid complexes, which define transcription factors, polymerases and more [187]. An example of such an approach produced a polyanionic DNA-linked hydrogel loaded with the cationic antimicrobial peptide L12 (LKKL)_3_ through charge complementation [188]. This material framework is composed of two unique DNA components—a Y-branched scaffold and L-shaped linker – that form a stable matrix upon hybridization at 95 °C (Figure 12d,f). Following electrostatic complexation with the cationic L12 peptide, a dendrimeric microparticle is created that can be tuned in size from 0.19–1.3 μm based on peptide concentration, with diameter positively correlating to peptide loading (0–320 µm). This hybrid co-assembly eliminated the cytotoxicity of L12 towards human dermal fibroblasts, while conserving its potent bactericidal activity towards methicillin-sensitive and -resistant *S. aureus*. Interestingly, an inverse behavior was observed for *E. coli*, where peptide loading into the DNA hydrogel effectively removed its activity towards this pathogen. This suggests that presentation of the AMP from the material scaffold altered its microbial specificity profile, thus representing potentially new materials-enabled approaches to improve the pathogen selectivity of therapeutics.

**Figure 13:**
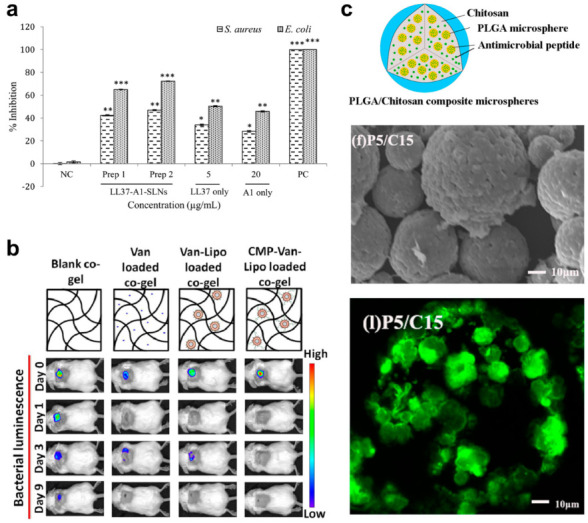
(**a**) Antibacterial activity of LL-37 and Serpin A1 towards *S. aureus* and *E. coli* enhanced by incorporation within a lipid carrier. Adapted with permission from [194]. Copyright 2016 American Chemical Society. (**b**) Enhanced in vivo antimicrobial activity of liposomal-vancomycin formulations encapsulated within a macroscopic collagen/fibrin gel (CMP). Reprinted from [195] with permission from Elsevier. (**c**) Schematic (top), SEM (middle), and fluorescence (bottom) images of composite microsphere scaffolds loaded with the antimicrobial peptide KSL-W. Image adapted from [199] under the Creative Commons Attribution 4.0 International License http://creativecommons.org/licenses/by/4.0/.

In contrast to the chemical versatility and serum instability of nucleic acids and carbohydrates, lipid-peptide co-assemblies represent a relative middle ground. While lipid carriers are a generally cytocompatibile platform that can be readily formulated with various biomolecular cargo [189], tailored chemical modifications and large-scale manufacture can be limited in comparison. However, a major advantage of liposomal-based drug carriers is the ability of membranolytic AMPs to intercalate into the bilayer to effectively turn ‘on’ their transition to a bioactive α-helical or β-sheet conformation [97,190,191,192]. This is in addition to the ability of liposomal particles to improve the systemic stability and circulation time of the loaded therapeutic. These advantages were leveraged by one of the original systems to adopt this strategy—a liposomal polymyxin B formulation—which played a transformative role in enabling the use of this therapeutic to combat outbreaks of Listeria [193]. Solid lipid nanoparticles have likewise been employed to deliver AMPs for wound healing and disinfection. For example, LL-37, an antimicrobial endogenous host defense peptide, and Serpin A1, an elastin inhibitor shown to improve wound healing, were co-formulated into a solid lipid nanoparticle to prevent bacterial infection of wounds and expedite closure [194]. Critically, the combination of LL-37 and Serpin A1 showed a ~20% improvement in *S. aureus* and *E. coli* growth inhibition, compared to the monotherapies, and was well tolerated by fibroblasts and keratinocytes at therapeutically relevant concentrations (cell viability >90%, Figure 13a). Sullivan and colleagues developed a combinatorial approach, in which a collagen/fibrin network was ultimately loaded with vancomycin-containing liposomes [195]. To do so, collagen mimetic peptides were hybridized to the outer surface, affording stable integration into the bulk hydrogel. As a result, liposomal and drug release from the system were drastically slowed and the antibacterial effects against *S. aureus* were delayed for up to 7 h after treatment. This proved to prolong the antimicrobial activity of the material in vivo, resulting in faster wound healing compared to controls and free drug (Figure 13b). Although outside the scope of this review, preparing liposomal formulations of AMPs have not been solely a novelty of infectious disease research, and have been exploited to repurpose these membranolytics into anti-tumor agents [196] and to protect agricultural crops from pathogens [197].

Finally, AMPs have also been successfully co-formulated with synthetic polymers. This approach leverages the generally weak immunogenicity of polymers to passivate the inflammatory responses often observed during preclinical use of cationic AMPs [198]. Poly(lactide-*co*-glycolide) (PLGA) has been widely used for such applications, and in one example has been combined with chitosan to prepare microspheres loaded with the antimicrobial decapeptide, KSL-W (KKVVFWVKFK-NH_2_, Figure 13c) [199]. Notably, these materials have shown antimicrobial efficacy against oral bacteria in vitro lasting over 80 days, suggesting their potential application to treat periodontitis. Polyethylene glycol (PEG) has also been used as a chemical spacer to ligate the enantiomeric AMP ^D^C^D^K^D^R^D^W^D^W^D^K^D^W^D^I^D^R^D^W-NH_2_ to the surface of a pre-formed hydrogel to prevent biofouling by *S. aureus* and *S. epidermis* [200]. Such strategies offer a convenient means by which antimicrobial coatings can be applied to implants and medical devices, including titanium [201].

## 4. Peptide-Facilitated Antimicrobial Technologies

Due to their natural polymorphism, peptides can play a wide range of functional and structural roles in antimicrobial materials. Here, three exemplary applications are reviewed to demonstrate unique strategies, employing peptides to enhance, modify or direct antimicrobial response in a variety of clinical applications.

### 4.1. Bioresponsive Delivery

**Figure 14:**
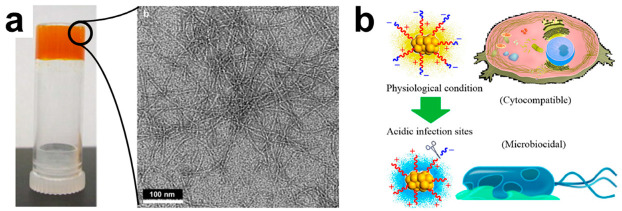
(**a**) Macroscopic (left) and TEM image (right) of a curcumin loaded MAX8 gel. Reprinted from [153] with permission from Elsevier. (**b**) Schematic image of site-specific cleavage of antimicrobial peptides from the gold nanocluster in acidic infection sites for targeted, bioresponsive activity. Reprinted with permission from [210]. Copyright 2019 American Chemical Society.

‘Smart’ biomaterials are designed to respond to pH, enzyme concentration, mechanical force and temperature, as well as a variety of other stimuli to temporally and spatially control their functional bioactivity [202,203,204]. Such materials provide unique control over cargo release in therapeutic [205] and diagnostic settings [206]. In one such example, MAX8 peptide hydrogels, originally developed for cell-delivery applications [207], were formulated with curcumin, a natural antibacterial molecule that is derived from turmeric (Figure 14a) [153,208], to sustainably release the agent as the peptide matrix is proteolytically degraded [209]. Leveraging the shear responsive nature of the MAX family peptides, these injectable and self-healing rigid materials show potential as fillers in weight-bearing joints to prevent post-surgical infections. In a separate work, a cysteine-terminated AMP (CysHHC10, H-CKRWWKWIRW-NH_2_) was ligated to the surface of gold nanoclusters to create a pH-responsive probe for bacterial biosensing [210]. The peptide’s primary amine groups were capped with anionic citraconyl motifs, which underwent autocleavage in acidic microenvironments, such as those found at infection sites, to re-expose the cationic side chains of the peptide to enable particle-bacteria binding (Figure 14b). Capping of the peptide amines with citraconyl also had the additional functionality of reducing degradation and clearance during systemic delivery. As a result, HHC10-Au nanoclusters showed broad spectrum activity towards *E. coli*, *P. aeruginosa*, *S. aureus* and *S. epidermidis* (MICs = 8–32 µg/mL), with minimal off-target hemolysis in vitro (<5% at therapeutic concentrations) and limited inflammation or tissue damage observed in vivo [210]. Self-assembled nanostructures templated by a WMR-derivative exhibit potent antimicrobial and, excitingly, anti-biofilm activity [211]. Here, the stability and degradation of the material in tissues could be tuned by controlling monomer concentration, ionic strength and pH; highlighting the potential to leverage the bioactive and bioresponsive nature of peptides to tailor the microenvironmentally regulated behavior of engineered materials.

### 4.2. Immunomodulatory Materials

**Figure 15:**
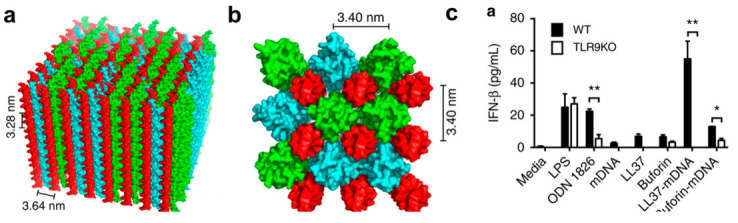
Nucleic acid helices complexed with (**a**) melittin and (**b**) LL-37. Peptide helices are shown in green (N to C polarity) or teal (C to N polarity), with dsDNA shown in red. (**c**) Induction of type 1 interferon production through TLR9-dependent mechanisms, highlighting the immunomodulatory capabilities of LL37-nucleic acid complexes. Image adapted from [213] under the Creative Commons Attribution 4.0 International License http://creativecommons.org/licenses/by/4.0/.

In contrast to interacting directly with bacterial cells to elicit antimicrobial responses, a select class of self-assembling peptide materials have been designed to elicit antibacterial effects by interfacing with the immune system. While monomeric peptides have been reported to induce immunomodulatory effects [182,183], fibrillar assemblies of antimicrobial peptides have also attracted significant interest based on their ability to interact with immunostimulatory nucleic acids and induce immune responses through a variety of molecular pathways [212]. Complexes containing the natural sequences LL-37, buforin and melittin can operate as Toll-like receptor (TLR) agonists, for example [182,213]. LL-37 in particular has been shown to have potent regulatory activity, both inhibitory and stimulatory, towards TLR 2/3/4, and serves as a chemoattractant for inflammatory cells [42]. Importantly, interactions of these peptide scaffolds with RNA and DNA can further differentiate their mechanistic responses by activating TLR3 and TLR9, respectively (Figure 15a–c). In tangential work, conjugation of SL9, a cytotoxic T lymphocyte epitope, to the peptide EAK16-II (AEAEAKAKAEAEAKAK), and co-assembly with the TLR7/8 agonists R848, led to the production of peptide-based nanofibrillar vaccines against HIV [214]. By tailoring their affinity for the TLR family, these materials have the potential for biochemical precision against target microorganisms that can be dictated by supramolecular architecture and identity of the immune cell(s) activated.

**Figure 16:**
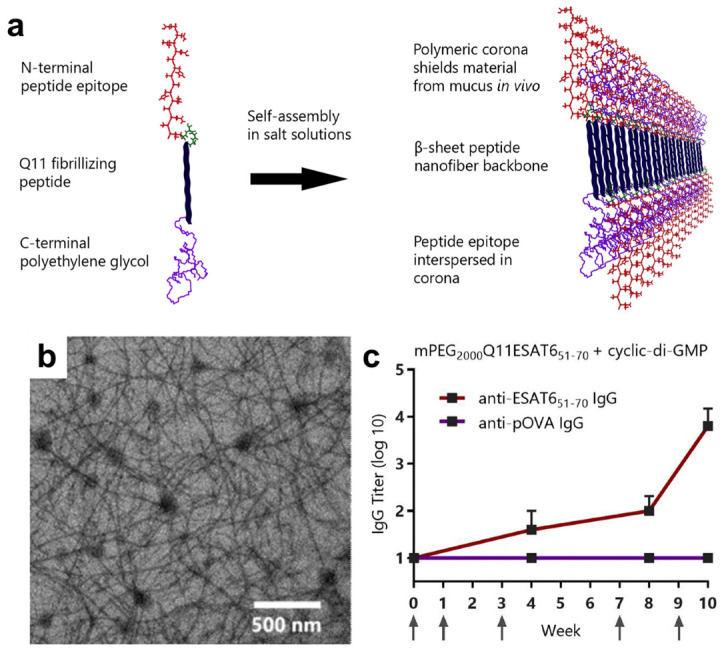
(**a**) Schematic demonstrating functionalized Q11 assembly. The Q11 backbone (navy) self assembles into a β-sheet fiber in ionic solutions. Immunogenic epitopes (red) and PEG motifs (purple) are functionalized to the peptide termini. (**b**) TEM image of self-assembled Q11 fibers conjugated with an ovalbumin epitope, OVA_323–339_. (**c**) Murine model IgG quantification demonstrating successful sublingual immunization against *M. tuberculosis* with PEG-Q11-ESAT conglomerate. Adapted from [220] with permission from Elsevier.

Collier, Chong and co-workers have developed nanofibrillar peptide vaccines consisting of a modified ovalbumin epitope, known for MHC-binding and T-cell activation, and the previously validated self-assembling sequence Q11 [215,216], to trigger multiple and redundant modes of CD4^+^ T cell activation [217]. In this study, partial knockdown of Myeloid differentiation primary response gene 88 (MyD88), the downstream adaptor of most TLRs, did not compromise activation of CD11c^+^ antigen presenting cells or CD4^+^ T cells, suggesting the self-assembled peptide nanofiber is capable of activating complementary MyD88-dependent and MyD88-independent signaling pathways. In separate work, intranasal delivery of Q11 nanofibers containing conserved H1N1 epitopes also elicited enhanced CD8^+^ T cell activation [218]. This hapten-like system has also been shown to be capable of T cell-independent B cell activation [219]. Sublingual vaccination was achieved by creating a hybrid complex, with each termini of the Q11 self-assembling sequence undergoing modification (Figure 16a,b) [220]. On one termini was conjugated an ESAT-6_51–70_ epitope, derived from an antigenic virulence factor of *M. tuberculosis* [221], and the other was functionalized with mPEG_2000_, or the entirely peptidic Pro-Ala-Ser motif, to prevent interactions with mucin and enable increased residence at the sublingual immunization site. This system produced robust antigen-specific IgG titers (10^2^–10^4^) in CBA/J mice one week after immunization (Figure 16c), with insignificant IFN-γ, IL-2 and IL-4 expression.

### 4.3. Active Targeting

Somewhat further removed from bioactivity in the context of cytotoxicity are peptides that provide active targeting for a compound or material. This is the basis of ligand-based peptide design, in which a known biochemical target is used to drive the development of novel therapeutics with high affinity [222]. The most well-known and widely utilized peptide targeting group is the RGD (Arg-Gly-Asp) motif. This tripeptide tag binds to integrins, and is therefore frequently used to target cancer cells [223,224,225]; however, this has also been applied to antimicrobial silver nanoparticles [226]. Chitin binding peptides have been shown to target fungal cell walls [227]. Such affinities allow for toxic peptides to be used at subtherapeutic concentrations as a microbial targeting motif. Simply displaying LL-37 from the surface of silica nanoparticles, for example, affords enhanced membrane localization and disruption of microbes [228]. Interestingly, the porosity of the particles dictated the dependence on co-delivery of free (unbound) LL-37. Surface charge, independent of porosity, was strongly correlated with binding efficiency, however it was noted that strongly bound LL-37 ultimately was unable to enact any bioactivity.

## 5. Future Directions

While work continues to discover new small molecule antibiotics, parallel efforts have begun to explore viral, protein and peptide biotherapeutics. Peptides have shown particular translational potential owing to their readily tailored design and unique mechanisms of action, ushering in an exciting intersection of biomaterials and microbiology. Table 1 below provides a summary of key examples discussed in this review.

These supramolecular peptide assemblies are often more proteolytically stable than the free monomer, elicit activity through multiple mechanisms unique from conventional therapeutics, offer increased potency, efficacy, and specificity, and provide simple methods to localize and target the desired bioactive agent. However, there remain several key barriers to the widespread clinical adoption of these technologies, including off-target cytotoxicity and poor pharmacokinetic properties. Novel methods of administering these peptide therapeutics (e.g., inhalable/oral formulations), and the incorporation of co-assembled excipients, is an active area of research to overcome these hurdles. Additional long-standing concerns with clinical translation of peptides, such as ease of screening and scalable manufacture, are being addressed through the continued development of multiplexed peptide libraries and microwave-assisted synthesis, respectively. Through such methods, chemists have made peptide therapeutics and materials viable candidates for future discovery ventures. In the coming years, interest is likely to increase as properties of physically cross-linked, bioinspired and biomimetic materials continue to be enhanced and understood. Sustained collaboration between biochemists, material scientists and infectious disease experts will determine the future viability of supramolecular peptide materials as novel weapons in the fight against drug-resistant microbes.

## Figures and Tables

**Table 1:** Summary of select peptide assemblies highlighted in this review.

Name (Sequence)	Morphology	Mechanism	Bioactive Conc.	Ref.
FF8 (KRRFFRRK)	β-Amyloid fibril	Bacteriolysis	25.6 μM	[73]
MAD1 (KRWHWWRRHWVVW-NH_2_)	β-Amyloid fibril	Bacteriolysis	2.5 μM	[74]
zp3 (GIIAGIIIKIKK-NH_2_)	Non β-Amyloid fibril	Bacteriolysis	8 μM	[94]
*Cg*-BigDef1 (GenBank: AEE92768.1)	Nanofibrillar net	Entrapment	0.3 μM	[109]
GL13K (GKIIKLKASLKLL-NH_2_)	Nanoribbon	Non-lytic/unknown	5 μg/mL	[115]
Diphenylalanine (FF)	Nanotube	Permeation/ depolarization	125 μg/mL	[71]
MARG1 (VKVKVRVKV^D^PPTKVKVRVKV-NH_2_)	Macroscopic gel	Electrostatic disruption	2 wt%	[128]
**Peptide**	**Co-Assembled Agent**	**Mechanism**	**Bioactive Response**	**Ref.**
^D^Leu–Phe–Phe	Ciprofloxacin	Combinatorial synergy	500–1000 fold enhanced toxicity	[148]
Poly(arginine-r-valine)-mannose	Zn^2+^	Combinatorial synergy	Conserved bacterial toxicity. Reduced toxicity	[165]
LL-37	Alginate	Enhanced delivery	Enhanced selectivity index. Prolonged release kinetics	[169]
L12	DNA scaffold and linker	Enhanced delivery	Induced selectivity	[188]
Collagen mimetic	Loaded liposome Collagen/fibrin	Enhanced delivery and retention	Improved in vivo efficacy	[195]
CysHHC10	Au nanoclusters	Autocleavable motif	pH responsive bioactivity. Minimized off target effects	[210]

## References

[1] Fleming A. (1929). Classics in Infectious Diseases: On the Antibacterial Action of Cultures of a Penicillium, With Special Reference to Their Use in the Isolation of B. Influenzae. Br. J. Exp. Pathol..

[2] Gaynes R. (2017). The discovery of penicillin—new insights after more than 75 years of clinical use. Emerg. Infect. Dis..

[3] Wood T.K., Knabel S.J., Kwan B.W. (2013). Bacterial persister cell formation and dormancy. Appl. Environ. Microbiol..

[4] Lewis K. (2012). Persister cells: Molecular mechanisms related to antibiotic tolerance. Antibiotic Resistance.

[5] Hoffman S.B. (2001). Mechanisms of Antibiotic Resistance. Compend. Contin. Educ. Pract. Vet..

[6] Lambert P.A. (2005). Bacterial resistance to antibiotics: Modified target sites. Adv. Drug Deliv. Rev..

[7] Dever L.A., Dermody T.S. (1991). Mechanisms of Bacterial Resistance to Antibiotics. Arch. Intern. Med..

[8] Soto S.M. (2013). Role of efflux pumps in the antibiotic resistance of bacteria embedded in a biofilm. Virulence.

[9] Blanco P., Hernando-Amado S., Reales-Calderon J., Corona F., Lira F., Alcalde-Rico M., Bernardini A., Sanchez M., Martinez J. (2016). Bacterial Multidrug Efflux Pumps: Much More Than Antibiotic Resistance Determinants. Microorganisms.

[10] van Duin D., Paterson D.L. (2016). Multidrug-Resistant Bacteria in the Community: Trends and Lessons Learned. Infect. Dis. Clin..

[11] Morley V.J., Woods R.J., Read A.F. (2019). Bystander Selection for Antimicrobial Resistance: Implications for Patient Health. Trends Microbiol..

[12] Norris J.S., Westwater C., Schofield D. (2000). Prokaryotic gene therapy to combat multidrug resistant bacterial infection. Gene Ther..

[13] Roach D.R., Donovan D.M. (2015). Antimicrobial bacteriophage-derived proteins and therapeutic applications. Bacteriophage.

[14] Sulakvelidze A., Alavidze Z., Morris J. (2001). Bacteriophage therapy. Antimicrob. Agents Chemother..

[15] Principi N., Silvestri E., Esposito S. (2019). Advantages and Limitations of Bacteriophages for the Treatment of Bacterial Infections. Front. Pharmacol..

[16] Lazzaro B.P., Zasloff M., Rolff J. (2020). Antimicrobial peptides: Application informed by evolution. Science.

[17] Brogden K.A. (2005). Antimicrobial peptides: Pore formers or metabolic inhibitors in bacteria?. Nat. Rev. Microbiol..

[18] Pfalzgraff A., Brandenburg K., Weindl G. (2018). Antimicrobial peptides and their therapeutic potential for bacterial skin infections and wounds. Front. Pharmacol..

[19] Zhang L., Gallo R.L. (2016). Antimicrobial peptides. Curr. Biol..

[20] Izadpanah A., Gallo R.L., Diego S. (2005). Antimicrobial peptides. J. Am. Acad. Dermatol..

[21] Opal S.M. (2016). Non-antibiotic treatments for bacterial diseases in an era of progressive antibiotic resistance. Crit. Care.

[22] Rex J.H., Fernandez Lynch H., Cohen I.G., Darrow J.J., Outterson K. (2019). Designing development programs for non-traditional antibacterial agents. Nat. Commun..

[23] Theuretzbacher U., Piddock L.J. (2019). V Review Non-traditional Antibacterial Therapeutic Options and Challenges. Cell Host Microbe.

[24] Kirienko N.V., Rahme L., Cho Y.H. (2019). Editorial: Beyond Antimicrobials: Non-traditional Approaches to Combating Multidrug-Resistant Bacteria. Front. Cell. Infect. Microbiol..

[25] Gaspar D., Veiga A.S., Castanho M.A.R.B. (2013). From antimicrobial to anticancer peptides. A review. Front. Microbiol..

[26] Henriques S.T., Melo M.N., Castanho M.A.R.B. (2006). Cell-penetrating peptides and antimicrobial peptides: How different are they?. Biochem. J..

[27] Hancock R.E.W., Scott M.G. (2000). The role of antimicrobial peptides in animal defenses. Proc. Natl. Acad. Sci. USA.

[28] Kumar P., Kizhakkedathu J.N., Straus S.K. (2018). Antimicrobial peptides: Diversity, mechanism of action and strategies to improve the activity and biocompatibility in vivo. Biomolecules.

[29] Guralp S.A., Murgha Y.E., Rouillard J.-M., Gulari E. (2013). From Design to Screening: A New Antimicrobial Peptide Discovery Pipeline. PLoS ONE.

[30] Fjell C.D., Hiss J.A., Hancock R.E.W., Schneider G. (2012). Designing antimicrobial peptides: Form follows function. Nat. Rev. Drug Discov..

[31] Tucker A.T., Leonard S.P., DuBois C.D., Knauf G.A., Cunningham A.L., Wilke C.O., Trent M.S., Davies B.W. (2018). Discovery of Next-Generation Antimicrobials through Bacterial Self-Screening of Surface-Displayed Peptide Libraries. Cell.

[32] Kulkarni K., Habila N., Del Borgo M.P., Aguilar M.I. (2019). Novel materials from the supramolecular self-assembly of short helical β3-peptide foldamers. Front. Chem..

[33] Wang J., Liu K., Xing R., Yan X. (2016). Peptide self-assembly: Thermodynamics and kinetics. Chem. Soc. Rev..

[34] Vanier G.S. (2013). Microwave-assisted solid-phase peptide synthesis based on the fmoc protecting group strategy (CEM). Methods Mol. Biol..

[35] Andersson L., Blomberg L., Flegel M., Lepsa L., Nilsson B., Verlander M. (2000). Large-scale synthesis of peptides. Pept. Sci..

[36] Bray B.L. (2003). Large-scale manufacture of peptide therapeutics by chemical synthesis. Nat. Rev. Drug Discov..

[37] Collier J.H., Segura T. (2011). Evolving the use of peptides as components of biomaterials. Biomaterials.

[38] Spicer C.D., Pashuck E.T., Stevens M.M. (2018). Achieving Controlled Biomolecule-Biomaterial Conjugation. Chem. Rev..

[39] Tang W., Becker M.L. (2014). “Click” reactions: A versatile toolbox for the synthesis of peptide-conjugates. Chem. Soc. Rev..

[40] Hancock R.E.W., Lehrer R. (1998). Cationic peptides: A new source of antibiotics. Trends Biotechnol..

[41] Brogden K.A., Ackermann M., McCray P.B., Tack B.F. (2003). Antimicrobial peptides in animals and their role in host defences. Int. J. Antimicrob. Agents.

[42] Mahlapuu M., Håkansson J., Ringstad L., Björn C. (2016). Antimicrobial peptides: An emerging category of therapeutic agents. Front. Cell. Infect. Microbiol..

[43] Hancock R.E.W. (1997). Peptide antibiotics. Lancet.

[44] Van’T Hof W., Veerman E.C.I., Heimerhorst E.J., Nieuw Amerongen A.V. (2001). Antimicrobial peptides: Properties and applicability. Biol. Chem..

[45] Shai Y. (2002). Mode of action of membrane active antimicrobial peptides. Biopolym. Pept. Sci. Sect..

[46] Wimley W.C., Hristova K. (2011). Antimicrobial peptides: Successes, challenges and unanswered questions. J. Membr. Biol..

[47] Powers J.P.S., Hancock R.E.W. (2003). The relationship between peptide structure and antibacterial activity. Peptides.

[48] Hancock R.E.W., Sahl H.G. (2006). Antimicrobial and host-defense peptides as new anti-infective therapeutic strategies. Nat. Biotechnol..

[49] Gordon Y.J., Romanowski E.G., McDermott A.M. (2005). A review of antimicrobial peptides and their therapeutic potential as anti-infective drugs. Curr. Eye Res..

[50] Marr A.K., Gooderham W.J., Hancock R.E. (2006). Antibacterial peptides for therapeutic use: Obstacles and realistic outlook. Curr. Opin. Pharmacol..

[51] Kagan B.L., Jang H., Capone R., Teran Arce F., Ramachandran S., Lal R., Nussinov R. (2012). Antimicrobial properties of amyloid peptides. Mol. Pharm..

[52] Zhang M., Zhao J., Zheng J. (2014). Molecular understanding of a potential functional link between antimicrobial and amyloid peptides. Soft Matter.

[53] Wang L., Liu Q., Chen J.C., Cui Y.X., Zhou B., Chen Y.X., Zhao Y.F., Li Y.M. (2012). Antimicrobial activity of human islet amyloid polypeptides: An insight into amyloid peptides’ connection with antimicrobial peptides. Biol. Chem..

[54] Kagan B.L. (2011). Antimicrobial amyloids?. Biophys. J..

[55] Hardy J.A., Higgins G.A. (1992). Alzheimer’s disease: The amyloid cascade hypothesis. Science.

[56] Tanzi R.E., Moir R.D., Wagner S.L. (2004). Clearance of Alzheimer’s Aβ peptide: The many roads to perdition. Neuron.

[57] Chiti F., Dobson C.M. (2006). Protein Misfolding, Functional Amyloid, and Human Disease. Annu. Rev. Biochem..

[58] Moir R.D., Lathe R., Tanzi R.E. (2018). The antimicrobial protection hypothesis of Alzheimer’s disease. Alzheimer’s Dement..

[59] Parady B. (2018). Innate Immune and Fungal Model of Alzheimer’s Disease. J. Alzheimer’s Dis. Reports.

[60] Spitzer P., Condic M., Herrmann M., Oberstein T.J., Scharin-Mehlmann M., Gilbert D.F., Friedrich O., Grömer T., Kornhuber J., Lang R. (2016). Amyloidogenic amyloid-β-peptide variants induce microbial agglutination and exert antimicrobial activity. Sci. Rep..

[61] Soscia S.J., Kirby J.E., Washicosky K.J., Tucker S.M., Ingelsson M., Hyman B., Burton M.A., Goldstein L.E., Duong S., Tanzi R.E. (2010). The Alzheimer’s Disease-Associated Amyloid β-Protein Is an Antimicrobial Peptide. PLoS ONE.

[62] Moir R.D., Tanzi R.E. (2019). Low Evolutionary Selection Pressure in Senescence Does Not Explain the Persistence of Aβ in the Vertebrate Genome. Front. Aging Neurosci..

[63] Harris F., Dennison S.R., Phoenix D.A. (2012). Aberrant action of amyloidogenic host defense peptides: A new paradigm to investigate neurodegenerative disorders?. FASEB J..

[64] Kandel N., Zheng T., Huo Q., Tatulian S.A. (2017). Membrane Binding and Pore Formation by a Cytotoxic Fragment of Amyloid β Peptide. J. Phys. Chem. B.

[65] Kumar D.K.V., Eimer W.A., Tanzi R.E., Moir R.D. (2016). Alzheimer’s disease: The potential therapeutic role of the natural antibiotic amyloid-β peptide. Neurodegener. Dis. Manag..

[66] Gosztyla M.L., Brothers H.M., Robinson S.R. (2018). Alzheimer’s Amyloid-β is an Antimicrobial Peptide: A Review of the Evidence. J. Alzheimer’s Dis..

[67] Peters C., Bascuñán D., Opazo C., Aguayo L.G. (2016). Differential Membrane Toxicity of amyloid-β Fragments by Pore Forming Mechanisms. J. Alzheimer’s Dis..

[68] Kumar D.K.V., Choi H.S., Washicosky K.J., Eimer W.A., Tucker S., Ghofrani J., Lefkowitz A., McColl G., Goldstein L.E., Tanzi R.E. (2016). Amyloid-β peptide protects against microbial infection in mouse and worm models of Alzheimer’s disease. Sci. Transl. Med..

[69] Giridharan V.V., Masud F., Petronilho F., Dal-Pizzol F., Barichello T. (2019). Infection-Induced Systemic Inflammation Is a Potential Driver of Alzheimer’s Disease Progression. Front. Aging Neurosci..

[70] Lee S., Trinh T.H.T., Yoo M., Shin J., Lee H., Kim J., Hwang E., Lim Y.B., Ryou C. (2019). Self-assembling peptides and their application in the treatment of diseases. Int. J. Mol. Sci..

[71] Schnaider L., Brahmachari S., Schmidt N.W., Mensa B., Shaham-Niv S., Bychenko D., Adler-Abramovich L., Shimon L.J.W., Kolusheva S., Degrado W.F. (2017). Self-assembling dipeptide antibacterial nanostructures with membrane disrupting activity. Nat. Commun..

[72] Hu Y., Lin R., Zhang P., Fern J., Cheetham A.G., Patel K., Schulman R., Kan C., Cui H. (2016). Electrostatic-driven lamination and untwisting of β-sheet assemblies. ACS Nano.

[73] Shen Z., Guo Z., Zhou L., Wang Y., Zhang J., Hu J., Zhang Y. (2020). Biomembrane induced in situ self-assembly of peptide with enhanced antimicrobial activity. Biomater. Sci..

[74] Simonson A.W., Mongia A.S., Aronson M.R., Alumasa J.N., Chan D.C., Bolotsky A., Ebrahimi A., Proctor E.A., Keiler K.C., Medina S.H. (2020). Pathogen-specific de novo antimicrobials engineered through membrane porin biomimicry. ChemRxiv.

[75] Xu L., Chou S., Wang J., Shao C., Li W., Zhu X., Shan A. (2015). Antimicrobial activity and membrane-active mechanism of tryptophan zipper-like β-hairpin antimicrobial peptides. Amino Acids.

[76] Panteleev P.V., Balandin S.V., Ovchinnikova T.V. (2017). Effect of Arenicins and Other β-Hairpin Antimicrobial Peptides on Pseudomonas Aeruginosa PAO1 Biofilms. Pharm. Chem. J..

[77] Miyata T., Tokunaga F., Yoneya T., Yoshikawa K., Iwanaga S., Niwa M., Takao T., Shimonishi Y. (1989). Antimicrobial peptides, isolated from horseshoe crab hemocytes, tachyplesin II, and polyphemusins I and II: Chemical structures and biological activity. J. Biochem..

[78] Edwards I.A., Elliott A.G., Kavanagh A.M., Zuegg J., Blaskovich M.A.T., Cooper M.A. (2016). Contribution of amphipathicity and hydrophobicity to the antimicrobial activity and cytotoxicity of β-hairpin peptides. ACS Infect. Dis..

[79] Wang C.K., King G.J., Conibear A.C., Ramos M.C., Chaousis S., Henriques S.T., Craik D.J. (2016). Mirror images of antimicrobial peptides provide reflections on their functions and amyloidogenic properties. J. Am. Chem. Soc..

[80] Wang C.K., Craik D.J. (2019). Toward Structure Determination of Disulfide-Rich Peptides Using Chemical Shift-Based Methods. J. Phys. Chem. B.

[81] Wang C.K., Northfield S.E., Huang Y.H., Ramos M.C., Craik D.J. (2016). Inhibition of tau aggregation using a naturally-occurring cyclic peptide scaffold. Eur. J. Med. Chem..

[82] Srinivas N., Jetter P., Ueberbacher B.J., Werneburg M., Zerbe K., Steinmann J., Van Der Meijden B., Bernardini F., Lederer A., Dias R.L.A. (2010). Peptidomimetic antibiotics target outer-membrane biogenesis in pseudomonas aeruginosa. Science.

[83] Panteleev P.V., Balandin S.V., Ivanov V.T., Ovchinnikova T.V. (2017). A Therapeutic Potential of Animal β-hairpin Antimicrobial Peptides. Curr. Med. Chem..

[84] Gour S., Kumar V., Singh A., Gadhave K., Goyal P., Pandey J., Giri R., Yadav J.K. (2019). Mammalian antimicrobial peptide protegrin-4 self assembles and forms amyloid-like aggregates: Assessment of its functional relevance. J. Pept. Sci..

[85] Jang H., Arce F.T., Mustata M., Ramachandran S., Capone R., Nussinov R., Lal R. (2011). Antimicrobial protegrin-1 forms amyloid-like fibrils with rapid kinetics suggesting a functional link. Biophys. J..

[86] Xu D., Chen W., Tobin-Miyaji Y.J., Sturge C.R., Yang S., Elmore B., Singh A., Pybus C., Greenberg D.E., Sellati T.J. (2018). Fabrication and Microscopic and Spectroscopic Characterization of Cytocompatible Self-Assembling Antimicrobial Nanofibers. ACS Infect. Dis..

[87] Xu D., Jiang L., Singh A., Dustin D., Yang M., Liu L., Lund R., Sellati T.J., Dong H. (2015). Designed supramolecular filamentous peptides: Balance of nanostructure, cytotoxicity and antimicrobial activity. Chem. Commun..

[88] Moore A.N., Hartgerink J.D. (2017). Self-Assembling Multidomain Peptide Nanofibers for Delivery of Bioactive Molecules and Tissue Regeneration. Acc. Chem. Res..

[89] Chen W., Yang S., Li S., Lang J.C., Mao C., Kroll P., Tang L., Dong H. (2019). Self-Assembled Peptide Nanofibers Display Natural Antimicrobial Peptides to Selectively Kill Bacteria without Compromising Cytocompatibility. ACS Appl. Mater. Interfaces.

[90] Chen W., Li S., Renick P., Yang S., Pandy N., Boutte C., Nguyen K.T., Tang L., Dong H. (2019). Bacterial acidity-triggered antimicrobial activity of self-assembling peptide nanofibers. J. Mater. Chem. B.

[91] Malishev R., Tayeb-Fligelman E., David S., Meijler M.M., Landau M., Jelinek R. (2018). Reciprocal Interactions between Membrane Bilayers and S. aureus PSMα3 Cross-α Amyloid Fibrils Account for Species-Specific Cytotoxicity. J. Mol. Biol..

[92] Tayeb-Fligelman E., Tabachnikov O., Moshe A., Goldshmidt-Tran O., Sawaya M.R., Coquelle N., Colletier J.P., Landau M. (2017). The cytotoxic Staphylococcus aureus PSMα3 reveals a cross-α amyloid-like fibril. Science.

[93] Salinas N., Colletier J.-P., Moshe A., Landau M. (2018). Extreme amyloid polymorphism in Staphylococcus aureus virulent PSMα peptides. Nat. Commun..

[94] Zeng P., Xu C., Cheng Q., Liu J., Gao W., Yang X., Wong K., Chen S., Chan K. (2019). Phenol-Soluble-Modulin-Inspired Amphipathic Peptides Have Bactericidal Activity against Multidrug-Resistant Bacteria. ChemMedChem.

[95] Zeth K., Sancho-Vaello E. (2017). The Human Antimicrobial Peptides Dermcidin and LL-37 Show Novel Distinct Pathways in Membrane Interactions. Front. Chem..

[96] Sancho-Vaello E., François P., Bonetti E.J., Lilie H., Finger S., Gil-Ortiz F., Gil-Carton D., Zeth K. (2017). Structural remodeling and oligomerization of human cathelicidin on membranes suggest fibril-like structures as active species. Sci. Rep..

[97] Aronson M.R., Simonson A.W., Orchard L.M., Llinás M., Medina S.H. (2018). Lipopeptisomes: Anticancer peptide-assembled particles for fusolytic oncotherapy. Acta Biomater..

[98] Sood R., Domanov Y., Pietiäinen M., Kontinen V.P., Kinnunen P.K.J. (2008). Binding of LL-37 to model biomembranes: Insight into target vs host cell recognition. Biochim. Biophys. Acta Biomembr..

[99] Shahmiri M., Enciso M., Adda C.G., Smith B.J., Perugini M.A., Mechler A. (2016). Membrane Core-Specific Antimicrobial Action of Cathelicidin LL-37 Peptide Switches between Pore and Nanofibre Formation. Sci. Rep..

[100] De Lorenzi E., Chiari M., Colombo R., Cretich M., Sola L., Vanna R., Gagni P., Bisceglia F., Morasso C., Lin J.S. (2017). Evidence that the human innate immune peptide LL-37 may be a binding partner of amyloid-β and inhibitor of fibril assembly. J. Alzheimer’s Dis..

[101] Chairatana P., Nolan E.M. (2014). Molecular basis for self-assembly of a human host-defense peptide that entraps bacterial pathogens. J. Am. Chem. Soc..

[102] Chairatana P., Nolan E.M. (2017). Human α-Defensin 6: A Small Peptide That Self-Assembles and Protects the Host by Entangling Microbes. Acc. Chem. Res..

[103] Schroeder B.O., Ehmann D., Precht J.C., Castillo P.A., Küchler R., Berger J., Schaller M., Stange E.F., Wehkamp J. (2015). Paneth cell α-defensin 6 (HD-6) is an antimicrobial peptide. Mucosal Immunol..

[104] Bergman P., Roan N.R., Römling U., Bevins C.L., Münch J. (2016). Amyloid formation: Functional friend or fearful foe?. J. Intern. Med..

[105] Sankaran-Walters S., Hart R., Dills C. (2017). Guardians of the gut: Enteric defensins. Front. Microbiol..

[106] Chu H., Pazgier M., Jung G., Nuccio S.P., Castillo P.A., De Jong M.F., Winter M.G., Winter S.E., Wehkamp J., Shen B. (2012). Human α-defensin 6 promotes mucosal innate immunity through self-assembled peptide nanonets. Science.

[107] Ehmann D., Wendler J., Koeninger L., Larsen I.S., Klag T., Berger J., Marette A., Schaller M., Stange E.F., Malek N.P. (2019). Paneth cell α-defensins HD-5 and HD-6 display differential degradation into active antimicrobial fragments. Proc. Natl. Acad. Sci. USA.

[108] Raschig J., Mailänder-Sánchez D., Berscheid A., Berger J., Strömstedt A.A., Courth L.F., Malek N.P., Brötz-Oesterhelt H., Wehkamp J. (2017). Ubiquitously expressed Human Beta Defensin 1 (hBD1) forms bacteria-entrapping nets in a redox dependent mode of action. PLoS Pathog..

[109] Loth K., Vergnes A., Barreto C., Voisin S.N., Meudal H., Da Silva J., Bressan A., Belmadi N., Bachère E., Aucagne V. (2019). The ancestral N-terminal domain of big defensins drives bacterially triggered assembly into antimicrobial nanonets. mBio.

[110] Stambuk F., Ojeda C., Schmitt P. Big Defensin ApBD1 from the scallop Argopecten purpuratus is an antimicrobial peptide which entraps bacteria through nanonets formation. bioRxiv.

[111] González R., Brokordt K., Cárcamo C.B., Coba de la Peña T., Oyanedel D., Mercado L., Schmitt P. (2017). Molecular characterization and protein localization of the antimicrobial peptide big defensin from the scallop Argopecten purpuratus after Vibrio splendidus challenge. Fish. Shellfish Immunol..

[112] Cao Y., Wang D., Zhou P., Zhao Y., Sun Y., Wang J. (2017). Influence of Conventional Surfactants on the Self-Assembly of a Bola Type Amphiphilic Peptide. Langmuir.

[113] Yang M., Yuan C., Shen G., Chang R., Xing R., Yan X. (2019). Cyclic dipeptide nanoribbons formed by dye-mediated hydrophobic self-assembly for cancer chemotherapy. J. Colloid Interface Sci..

[114] Tao K., Levin A., Adler-Abramovich L., Gazit E. (2016). Fmoc-modified amino acids and short peptides: Simple bio-inspired building blocks for the fabrication of functional materials. Chem. Soc. Rev..

[115] Abdolhosseini M., Nandula S.R., Song J., Hirt H., Gorr S.U. (2012). Lysine substitutions convert a bacterial-agglutinating peptide into a bactericidal peptide that retains anti-lipopolysaccharide activity and low hemolytic activity. Peptides.

[116] Harmouche N., Aisenbrey C., Porcelli F., Xia Y., Nelson S.E.D., Chen X., Raya J., Vermeer L., Aparicio C., Veglia G. (2017). Solution and Solid-State Nuclear Magnetic Resonance Structural Investigations of the Antimicrobial Designer Peptide GL13K in Membranes. Biochemistry.

[117] Hirt H., Hall J.W., Larson E., Gorr S.-U. (2018). A D-enantiomer of the antimicrobial peptide GL13K evades antimicrobial resistance in the Gram positive bacteria Enterococcus faecalis and Streptococcus gordonii. PLoS ONE.

[118] Ye Z., Zhu X., Acosta S., Kumar D., Sang T., Aparicio C. (2019). Self-assembly dynamics and antimicrobial activity of all l- and d-amino acid enantiomers of a designer peptide. Nanoscale.

[119] Porter S.L., Coulter S.M., Pentlavalli S., Thompson T.P., Laverty G. (2018). Self-assembling diphenylalanine peptide nanotubes selectively eradicate bacterial biofilm infection. Acta Biomater..

[120] Yang K., Han Q., Chen B., Zheng Y., Zhang K., Li Q., Wang J. (2018). Antimicrobial hydrogels: Promising materials for medical application. Int. J. Nanomed..

[121] Ng V.W.L., Chan J.M.W., Sardon H., Ono R.J., García J.M., Yang Y.Y., Hedrick J.L. (2014). Antimicrobial hydrogels: A new weapon in the arsenal against multidrug-resistant infections. Adv. Drug Deliv. Rev..

[122] Salomé Veiga A., Schneider J.P. (2013). Antimicrobial hydrogels for the treatment of infection. Biopolymers.

[123] Veiga A.S., Sinthuvanich C., Gaspar D., Franquelim H.G., Castanho M.A.R.B., Schneider J.P. (2012). Arginine-rich self-assembling peptides as potent antibacterial gels. Biomaterials.

[124] Salick D.A., Kretsinger J.K., Pochan D.J., Schneider J.P. (2007). Inherent antibacterial activity of a peptide-based β-hairpin hydrogel. J. Am. Chem. Soc..

[125] Kretsinger J.K., Haines L.A., Ozbas B., Pochan D.J., Schneider J.P. (2005). Cytocompatibility of self-assembled β-hairpin peptide hydrogel surfaces. Biomaterials.

[126] Nagy-Smith K., Moore E., Schneider J., Tycko R., DeGrado W.F. (2015). Molecular structure of monomorphic peptide fibrils within a kinetically trapped hydrogel network. Proc. Natl. Acad. Sci. USA.

[127] Nagy-Smith K., Beltramo P.J., Moore E., Tycko R., Furst E.M., Schneider J.P. (2017). Molecular, Local, and Network-Level Basis for the Enhanced Stiffness of Hydrogel Networks Formed from Coassembled Racemic Peptides: Predictions from Pauling and Corey. ACS Cent. Sci..

[128] Salick D.A., Pochan D.J., Schneider J.P. (2009). Design of an Injectable β-Hairpin Peptide Hydrogel That Kills Methicillin-Resistant Staphylococcus aureus. Adv. Mater..

[129] Yan C., Altunbas A., Yucel T., Nagarkar R.P., Schneider J.P., Pochan D.J. (2010). Injectable solid hydrogel: Mechanism of shear-thinning and immediate recovery of injectable β-hairpin peptide hydrogels. Soft Matter.

[130] Jiang L., Xu D., Sellati T.J., Dong He Dong H. (2015). Self-assembly of cationic multidomain peptide hydrogels: Supramolecular nanostructure and rheological properties dictate antimicrobial activity. Nanoscale.

[131] Bai J., Chen C., Wang J., Zhang Y., Cox H., Zhang J., Wang Y., Penny J., Waigh T., Lu J.R. (2016). Enzymatic Regulation of Self-Assembling Peptide A9K2 Nanostructures and Hydrogelation with Highly Selective Antibacterial Activities. ACS Appl. Mater. Interfaces.

[132] Sarkar B., Siddiqui Z., Nguyen P.K., Dube N., Fu W., Park S., Jaisinghani S., Paul R., Kozuch S.D., Deng D. (2019). Membrane-Disrupting Nanofibrous Peptide Hydrogels. ACS Biomater. Sci. Eng..

[133] McCloskey A.P., Draper E.R., Gilmore B.F., Laverty G. (2017). Ultrashort self-assembling Fmoc-peptide gelators for anti-infective biomaterial applications. J. Pept. Sci..

[134] Raeburn J., Cardoso A.Z., Adams D.J. (2013). The importance of the self-assembly process to control mechanical properties of low molecular weight hydrogels. Chem. Soc. Rev..

[135] Mahler A., Reches M., Rechter M., Cohen S., Gazit E. (2006). Rigid, Self-Assembled Hydrogel Composed of a Modified Aromatic Dipeptide. Adv. Mater..

[136] Tang C., Smith A.M., Collins R.F., Ulijn R.V., Saiani A. (2009). Fmoc-Diphenylalanine Self-Assembly Mechanism Induces Apparent pK a Shifts. Langmuir.

[137] Hughes M., Debnath S., Knapp C.W., Ulijn R.V. (2013). Antimicrobial properties of enzymatically triggered self-assembling aromatic peptide amphiphiles. Biomater. Sci..

[138] Gahane A.Y., Ranjan P., Singh V., Sharma R.K., Sinha N., Sharma M., Chaudhry R., Thakur A.K. (2018). Fmoc-phenylalanine displays antibacterial activity against Gram-positive bacteria in gel and solution phases. Soft Matter.

[139] Xie Y.Y., Zhang Y.W., Qin X.T., Liu L.P., Wahid F., Zhong C., Jia S.R. (2020). Structure-Dependent Antibacterial Activity of Amino Acid-Based Supramolecular Hydrogels. Colloids Surfaces B Biointerfaces.

[140] Shi J., Votruba A.R., Farokhzad O.C., Langer R. (2010). Nanotechnology in drug delivery and tissue engineering: From discovery to applications. Nano Lett..

[141] Pizzolato-Cezar L.R., Okuda-Shinagawa N.M., Machini M.T. (2019). Combinatory Therapy Antimicrobial Peptide-Antibiotic to Minimize the Ongoing Rise of Resistance. Front. Microbiol..

[142] Grassi L., Maisetta G., Esin S., Batoni G. (2017). Combination Strategies to Enhance the Efficacy of Antimicrobial Peptides against Bacterial Biofilms. Front. Microbiol..

[143] Wu X., Li Z., Li X., Tian Y., Fan Y., Yu C., Zhou B., Liu Y., Xiang R., Yang L. (2017). Synergistic effects of antimicrobial peptide DP7 combined with antibiotics against multidrug-resistant bacteria. Drug Des. Devel. Ther..

[144] Zeng P., Xu C., Liu C., Liu J., Cheng Q., Gao W., Yang X., Chen S., Chan K.F., Wong K.Y. (2020). De Novo Designed Hexadecapeptides Synergize Glycopeptide Antibiotics Vancomycin and Teicoplanin against Pathogenic Klebsiella pneumoniae via Disruption of Cell Permeability and Potential. ACS Appl. Bio Mater..

[145] Pletzer D., Mansour S.C., Hancock R.E.W. (2018). Synergy between conventional antibiotics and anti-biofilm peptides in a murine, sub-cutaneous abscess model caused by recalcitrant ESKAPE pathogens. PLoS Pathog..

[146] Aqeele E., Abkooh E. (2019). Determination of the Effective Dose of Curcumin alone and in Combination with Antimicrobial Peptide CM11 on Promastigote Forms of Iranian Strain of L. major (MRHO/IR/75/ER). Arch. Razi Inst..

[147] Marchesan S., Qu Y., Waddington L.J., Easton C.D., Glattauer V., Lithgow T.J., McLean K.M., Forsythe J.S., Hartley P.G. (2013). Self-assembly of ciprofloxacin and a tripeptide into an antimicrobial nanostructured hydrogel. Biomaterials.

[148] Gahane A.Y., Singh V., Kumar A., Kumar Thakur A. (2020). Development of mechanism-based antibacterial synergy between Fmoc-phenylalanine hydrogel and aztreonam. Biomater. Sci..

[149] Vegners R., Shestakova I., Kalvinsh I., Ezzell R.M., Janmey P.A. (1995). Use of a gel-forming dipeptide derivative as a carrier for antigen presentation. J. Pept. Sci..

[150] Thota C.K., Yadav N., Chauhan V.S. (2016). A novel highly stable and injectable hydrogel based on a conformationally restricted ultrashort peptide. Sci. Rep..

[151] Guler M.O., Claussen R.C., Stupp S.I. (2005). Encapsulation of pyrene within self-assembled peptide amphiphile nanofibers. J. Mater. Chem..

[152] Nagai Y., Unsworth L.D., Koutsopoulos S., Zhang S. (2006). Slow release of molecules in self-assembling peptide nanofiber scaffold. J. Control. Release.

[153] Altunbas A., Lee S.J., Rajasekaran S.A., Schneider J.P., Pochan D.J. (2011). Encapsulation of curcumin in self-assembling peptide hydrogels as injectable drug delivery vehicles. Biomaterials.

[154] McCloskey A.P., Gilmore S.M., Zhou J., Draper E.R., Porter S., Gilmore B.F., Xu B., Laverty G. (2016). Self-assembling ultrashort NSAID-peptide nanosponges: Multifunctional antimicrobial and anti-inflammatory materials. RSC Adv..

[155] Li X., Fan R., Tong A., Yang M., Deng J., Zhou L., Zhang X., Guo G. (2015). In situ gel-forming AP-57 peptide delivery system for cutaneous wound healing. Int. J. Pharm..

[156] Augst A.D., Kong H.J., Mooney D.J. (2006). Alginate Hydrogels as Biomaterials. Macromol. Biosci..

[157] Pasparakis G., Bouropoulos N. (2006). Swelling studies and in vitro release of verapamil from calcium alginate and calcium alginate-chitosan beads. Int. J. Pharm..

[158] Mikula K., Skrzypczak D., Ligas B., Witek-Krowiak A. (2019). Preparation of hydrogel composites using Ca2+ and Cu2+ ions as crosslinking agents. SN Appl. Sci..

[159] Chen W.-Y., Chang H.-Y., Lu J.-K., Huang Y.-C., Harroun S.G., Tseng Y.-T., Li Y.-J., Huang C.-C., Chang H.-T. (2015). Self-Assembly of Antimicrobial Peptides on Gold Nanodots: Against Multidrug-Resistant Bacteria and Wound-Healing Application. Adv. Funct. Mater..

[160] Rai A., Pinto S., Velho T.R., Ferreira A.F., Moita C., Trivedi U., Evangelista M., Comune M., Rumbaugh K.P., Simões P.N. (2016). One-step synthesis of high-density peptide-conjugated gold nanoparticles with antimicrobial efficacy in a systemic infection model. Biomaterials.

[161] Huang C.-C., Yang Z., Lee K.-H., Chang H.-T. (2007). Synthesis of Highly Fluorescent Gold Nanoparticles for Sensing Mercury(II). Angew. Chemie Int. Ed..

[162] Yang T.-Q., Peng B., Shan B.-Q., Zong Y.-X., Jiang J.-G., Wu P., Zhang K. (2020). Origin of the Photoluminescence of Metal Nanoclusters: From Metal-Centered Emission to Ligand-Centered Emission. Nanomaterials.

[163] Simon T., Wu C.S., Liang J.C., Cheng C., Ko F.H. (2016). Facile synthesis of a biocompatible silver nanoparticle derived tripeptide supramolecular hydrogel for antibacterial wound dressings. New J. Chem..

[164] Song J., Yuan C., Jiao T., Xing R., Yang M., Adams D.J., Yan X. (2020). Multifunctional Antimicrobial Biometallohydrogels Based on Amino Acid Coordinated Self-Assembly. Small.

[165] Panja S., Bharti R., Dey G., Lynd N.A., Chattopadhyay S. (2019). Coordination-assisted self-assembled polypeptide nanogels to selectively combat bacterial infection. ACS Appl. Mater. Interfaces.

[166] Pasquet J., Chevalier Y., Pelletier J., Couval E., Bouvier D., Bolzinger M.A. (2014). The contribution of zinc ions to the antimicrobial activity of zinc oxide. Colloids Surfaces A Physicochem. Eng. Asp..

[167] Chan C., Burrows L.L., Deber C.M. (2004). Helix induction in antimicrobial peptides by alginate in biofilms. J. Biol. Chem..

[168] Lin Z., Wu T., Wang W., Li B., Wang M., Chen L., Xia H., Zhang T. (2019). Biofunctions of antimicrobial peptide-conjugated alginate/hyaluronic acid/collagen wound dressings promote wound healing of a mixed-bacteria-infected wound. Int. J. Biol. Macromol..

[169] Toppazzini M., Coslovi A., Boschelle M., Marsich E., Benincasa M., Gennaro R., Paoletti S. (2011). Can the interaction between the antimicrobial peptide LL-37 and alginate be exploited for the formulation of new biomaterials with antimicrobial properties?. Carbohydr. Polym..

[170] Mateescu M., Baixe S., Garnier T., Jierry L., Ball V., Haikel Y., Metz-Boutigue M.H., Nardin M., Schaaf P., Etienne O. (2015). Antibacterial peptide-based gel for prevention of medical implanted-device infection. PLoS ONE.

[171] Pereira P., Pedrosa S.S., Correia A., Lima C.F., Olmedo M.P., González-Fernández Á., Vilanova M., Gama F.M. (2015). Biocompatibility of a self-assembled glycol chitosan nanogel. Toxicol. In Vitro.

[172] Wu C., Wu T., Fang Z., Zheng J., Xu S., Chen S., Hu Y., Ye X. (2016). Formation, characterization and release kinetics of chitosan/γ-PGA encapsulated nisin nanoparticles. RSC Adv..

[173] He Y., Jin Y., Wang X., Yao S., Li Y., Wu Q., Ma G., Cui F., Liu H. (2018). An antimicrobial peptide-loaded gelatin/chitosan nanofibrous membrane fabricated by sequential layer-by-layer electrospinning and electrospraying techniques. Nanomaterials.

[174] Water J.J., Kim Y., Maltesen M.J., Franzyk H., Foged C., Nielsen H.M. (2015). Hyaluronic acid-based nanogels produced by microfluidics-facilitated self-assembly improves the safety profile of the cationic host defense peptide novicidin. Pharm. Res..

[175] Silva J.P., Gonçalves C., Costa C., Sousa J., Silva-Gomes R., Castro A.G., Pedrosa J., Appelberg R., Gama F.M. (2016). Delivery of LLKKK18 loaded into self-assembling hyaluronic acid nanogel for tuberculosis treatment. J. Control. Release.

[176] Simonson A.W., Lawanprasert A., Goralski T.D.P., Keiler K.C., Medina S.H. (2019). Bioresponsive peptide-polysaccharide nanogels—A versatile delivery system to augment the utility of bioactive cargo. Nanomedicine.

[177] Huang R., Qi W., Feng L., Su R., He Z. (2011). Self-assembling peptide-polysaccharide hybrid hydrogel as a potential carrier for drug delivery. Soft Matter.

[178] Khoushab F., Jaruseranee N., Tanthanuch W., Yamabhai M. (2012). Formation of chitin-based nanomaterials using a chitin-binding peptide selected by phage-display. Int. J. Biol. Macromol..

[179] Turner J., Cho Y., Dinh N.N., Waring A.J., Lehrer R.I. (1998). Activities of LL-37, a cathelin-associated antimicrobial peptide of human neutrophils. Antimicrob. Agents Chemother..

[180] Lequeux I., Ducasse E., Jouenne T., Thebault P. (2014). Addition of antimicrobial properties to hyaluronic acid by grafting of antimicrobial peptide. Eur. Polym. J..

[181] Chu T.W., Feng J., Yang J., Kopeček J. (2015). Hybrid polymeric hydrogels via peptide nucleic acid (PNA)/DNA complexation. J. Control. Release.

[182] Lee E.Y., Takahashi T., Curk T., Dobnikar J., Gallo R.L., Wong G.C.L. (2017). Crystallinity of Double-Stranded RNA-Antimicrobial Peptide Complexes Modulates Toll-Like Receptor 3-Mediated Inflammation. ACS Nano.

[183] Macleod T., Ward J., Alase A.A., Bridgewood C., Wittmann M., Stonehouse N.J. (2019). Antimicrobial Peptide LL-37 Facilitates Intracellular Uptake of RNA Aptamer Apt 21-2 Without Inducing an Inflammatory or Interferon Response. Front. Immunol..

[184] Mondal S., Adler-Abramovich L., Lampel A., Bram Y., Lipstman S., Gazit E. (2015). Formation of functional super-helical assemblies by constrained single heptad repeat. Nat. Commun..

[185] Seeman N.C., Sleiman H.F. (2017). DNA nanotechnology. Nat. Rev. Mater..

[186] Li Z., Wang J., Li Y., Liu X., Yuan Q. (2018). Self-assembled DNA nanomaterials with highly programmed structures and functions. Mater. Chem. Front..

[187] Senyürek I., Klein G., Kalbacher H., Deeg M., Schittek B. (2010). Peptides derived from the human laminin α4 and α5 chains exhibit antimicrobial activity. Peptides.

[188] Obuobi S., Tay H.K.L., Tram N.D.T., Selvarajan V., Khara J.S., Wang Y., Ee P.L.R. (2019). Facile and efficient encapsulation of antimicrobial peptides via crosslinked DNA nanostructures and their application in wound therapy. J. Control. Release.

[189] Müller R.H., Runge S., Ravelli V., Mehnert W., Thünemann A.F., Souto E.B. (2006). Oral bioavailability of cyclosporine: Solid lipid nanoparticles (SLN^®^) versus drug nanocrystals. Int. J. Pharm..

[190] Avrahami D., Shai Y. (2003). Bestowing Antifungal and Antibacterial Activities by Lipophilic Acid Conjugation to D, L-Amino Acid-Containing Antimicrobial Peptides: A Plausible Mode of Action. Biochemistry.

[191] Dong W., Liu Z., Sun L., Wang C., Guan Y., Mao X., Shang D. (2018). Antimicrobial activity and self-assembly behavior of antimicrobial peptide chensinin-1b with lipophilic alkyl tails. Eur. J. Med. Chem..

[192] Cummings J.E., Vanderlick T.K. (2007). Aggregation and hemi-fusion of anionic vesicles induced by the antimicrobial peptide cryptdin-4. Biochim. Biophys. Acta Biomembr..

[193] McAllister S.M., Alpar H.O., Brown M.R.W. (1999). Antimicrobial properties of liposomal polymyxin B. J. Antimicrob. Chemother..

[194] Fumakia M., Ho E.A. (2016). Nanoparticles encapsulated with LL37 and serpin A1 promotes wound healing and synergistically enhances antibacterial activity. Mol. Pharm..

[195] Thapa R.K., Kiick K.L., Sullivan M.O. (2020). Encapsulation of collagen mimetic peptide-tethered vancomycin liposomes in collagen-based scaffolds for infection control in wounds. Acta Biomater..

[196] Zhang Q., Tang J., Ran R., Liu Y., Zhang Z., Gao H., He Q. (2016). Development of an anti-microbial peptide-mediated liposomal delivery system: A novel approach towards pH-responsive anti-microbial peptides. Drug Deliv..

[197] Velasquez Guzman J.C., Basu S., Rabara R., Huynh L.K., Basu G.C., Nguyen H.B., Gupta G. (2018). Liposome Delivery System of Antimicrobial Peptides against Huanglongbing (HLB) Citrus Disease. Biophys. J..

[198] Mowery B.P., Lee S.E., Kissounko D.A., Epand R.F., Epand R.M., Weisblum B., Stahl S.S., Gellman S.H. (2007). Mimicry of Antimicrobial Host-Defense Peptides by Random Copolymers The eukaryotic innate immune response to bacterial infection includes the production of peptides that kill prokaryotic invaders. J. Am. Chem. Soc..

[199] Li Y., Na R., Wang X., Liu H., Zhao L., Sun X., Ma G., Cui F. (2017). Fabrication of Antimicrobial Peptide-Loaded PLGA/Chitosan Composite Microspheres for Long-Acting Bacterial Resistance. Molecules.

[200] Cleophas R.T.C., Sjollema J., Busscher H.J., Kruijtzer J.A.W., Liskamp R.M.J. (2014). Characterization and activity of an immobilized antimicrobial peptide containing bactericidal PEG-hydrogel. Biomacromolecules.

[201] Gabriel M., Nazmi K., Veerman E.C., Amerongen A.V.N., Zentner A. (2006). Preparation of LL-37-grafted titanium surfaces with bactericidal activity. Bioconjug. Chem..

[202] Lu Y., Aimetti A.A., Langer R., Gu Z. (2016). Bioresponsive materials. Nat. Rev. Mater..

[203] Zhao F., Ma M.L., Xu B. (2009). Molecular hydrogels of therapeutic agents. Chem. Soc. Rev..

[204] Mart R.J., Osborne R.D., Stevens M.M., Ulijn R.V. (2006). Peptide-based stimuli-responsive biomaterials. Soft Matter.

[205] Webber M.J., Matson J.B., Tamboli V.K., Stupp S.I. (2012). Controlled release of dexamethasone from peptide nanofiber gels to modulate inflammatory response. Biomaterials.

[206] Lu S., Guo X., Zou M., Zheng Z., Li Y., Li X., Li L., Wang H. (2020). Bacteria-Instructed In Situ Aggregation of AuNPs with Enhanced Photoacoustic Signal for Bacterial Infection Bioimaging. Adv. Healthc. Mater..

[207] Haines-Butterick L., Rajagopal K., Branco M., Salick D., Rughani R., Pilarz M., Lamm M.S., Pochan D.J., Schneider J.P. (2007). Controlling hydrogelation kinetics by peptide design for three-dimensional encapsulation and injectable delivery of cells. Proc. Natl. Acad. Sci. USA.

[208] Shlar I., Droby S., Choudhary R., Rodov V. (2017). The mode of antimicrobial action of curcumin depends on the delivery system: Monolithic nanoparticles vs. supramolecular inclusion complex. RSC Adv..

[209] Li Z., He C., Yuan B., Dong X., Chen X. (2017). Injectable Polysaccharide Hydrogels as Biocompatible Platforms for Localized and Sustained Delivery of Antibiotics for Preventing Local Infections. Macromol. Biosci..

[210] Pranantyo D., Liu P., Zhong W., Kang E.T., Chan-Park M.B. (2019). Antimicrobial Peptide-Reduced Gold Nanoclusters with Charge-Reversal Moieties for Bacterial Targeting and Imaging. Biomacromolecules.

[211] Lombardi L., Shi Y., Falanga A., Galdiero E., De Alteriis E., Franci G., Chourpa I., Azevedo H.S., Galdiero S. (2019). Enhancing the Potency of Antimicrobial Peptides through Molecular Engineering and Self-Assembly. Biomacromolecules.

[212] Wen Y., Collier J.H. (2015). Supramolecular peptide vaccines: Tuning adaptive immunity. Curr. Opin. Immunol..

[213] Lee E.Y., Zhang C., Di Domizio J., Jin F., Connell W., Hung M., Malkoff N., Veksler V., Gilliet M., Ren P. (2019). Helical antimicrobial peptides assemble into protofibril scaffolds that present ordered dsDNA to TLR9. Nat. Commun..

[214] Ding Y., Liu J., Lu S., Igweze J., Xu W., Kuang D., Zealey C., Liu D., Gregor A., Bozorgzad A. (2016). Self-assembling peptide for co-delivery of HIV-1 CD8+ T cells epitope and Toll-like receptor 7/8 agonists R848 to induce maturation of monocyte derived dendritic cell and augment polyfunctional cytotoxic T lymphocyte (CTL) response. J. Control. Release.

[215] Collier J.H., Messersmith P.B. (2003). Enzymatic modification of self-assembled peptide structures with tissue transglutaminase. Bioconjug. Chem..

[216] Hudalla G.A., Modica J.A., Tian Y.F., Rudra J.S., Chong A.S., Sun T., Mrksich M., Collier J.H. (2013). A Self-Adjuvanting Supramolecular Vaccine Carrying a Folded Protein Antigen. Adv. Healthc. Mater..

[217] Si Y., Wen Y., Chen J., Pompano R.R., Han H., Collier J.H., Chong A.S. (2018). MyD88 in antigen-presenting cells is not required for CD4+ T-cell responses during peptide nanofiber vaccination. Medchemcomm.

[218] Si Y., Wen Y., Kelly S.H., Chong A.S., Collier J.H. (2018). Intranasal delivery of adjuvant-free peptide nanofibers elicits resident CD8+ T cell responses. J. Control. Release.

[219] Rudra J.S., Tian Y.F., Jung J.P., Collier J.H. (2010). A self-assembling peptide acting as an immune adjuvant. Proc. Natl. Acad. Sci. USA.

[220] Kelly S.H., Wu Y., Varadhan A.K., Curvino E.J., Chong A.S., Collier J.H. (2020). Enabling sublingual peptide immunization with molecular self-assemblies. Biomaterials.

[221] Sreejit G., Ahmed A., Parveen N., Jha V., Valluri V.L., Ghosh S., Mukhopadhyay S. (2014). The ESAT-6 Protein of Mycobacterium tuberculosis Interacts with Beta-2-Microglobulin (β2M) Affecting Antigen Presentation Function of Macrophage. PLoS Pathog..

[222] Gruber C.W., Muttenthaler M., Freissmuth M. (2010). Ligand-Based Peptide Design and Combinatorial Peptide Libraries to Target G Protein-Coupled Receptors. Curr. Pharm. Des..

[223] Medina S.H., Michie M.S., Miller S.E., Schnermann M.J., Schneider J.P. (2017). Fluorous Phase-Directed Peptide Assembly Affords Nano-Peptisomes Capable of Ultrasound-Triggered Cellular Delivery. Angew. Chemie Int. Ed..

[224] Sloand J.N., Nguyen T.T., Zinck S.A., Cook E.C., Zimudzi T.J., Showalter S.A., Glick A.B., Simon J.C., Medina S.H. (2020). Ultrasound-Guided Cytosolic Protein Delivery via Transient Fluorous Masks. ACS Nano.

[225] Asati S., Pandey V., Soni V. (2019). RGD Peptide as a Targeting Moiety for Theranostic Purpose: An Update Study. Int. J. Pept. Res. Ther..

[226] Di Pietro P., Zaccaro L., Comegna D., Del Gatto A., Saviano M., Snyders R., Cossement D., Satriano C., Rizzarelli E. (2016). Silver nanoparticles functionalized with a fluorescent cyclic RGD peptide: A versatile integrin targeting platform for cells and bacteria. RSC Adv..

[227] Pushpanathan M., Rajendhran J., Jayashree S., Sundarakrishnan B., Jayachandran S., Gunasekaran P. (2012). Identification of a Novel Antifungal Peptide with Chitin-Binding Property from Marine Metagenome. Protein Pept. Lett..

[228] Braun K., Pochert A., Lindén M., Davoudi M., Schmidtchen A., Nordström R., Malmsten M. (2016). Membrane interactions of mesoporous silica nanoparticles as carriers of antimicrobial peptides. J. Colloid Interface Sci..

